# Nanostructure and Photovoltaic Potential of Plasmonic Nanofibrous Active Layers

**DOI:** 10.1002/smll.202409269

**Published:** 2024-11-22

**Authors:** Ryan M. Schofield, Barbara M. Maciejewska, Karim A. Elmestekawy, Jack M. Woolley, George. T. Tebbutt, Mohsen Danaie, Christopher S. Allen, Laura M. Herz, Hazel E. Assender, Nicole Grobert

**Affiliations:** ^1^ Department of Materials University of Oxford Parks Road Oxford OX1 3PH UK; ^2^ Department of Physics University of Oxford Clarendon Laboratory Parks Road Oxford OX1 3PU UK; ^3^ Department of Physics University of Warwick Gibbet Hill Road Coventry CV4 7AL UK; ^4^ Electron Physical Science Imaging Centre Diamond Light Source Didcot OX11 0DE UK

**Keywords:** charge dynamics, crystallite alignment, electrospinning, exciton dissociation, nanofibers, photovoltaics

## Abstract

Nanofibrous active layers offer hierarchical control over molecular structure, and the size and distribution of electron donor:acceptor domains, beyond conventional organic photovoltaic architectures. This structure is created by forming donor pathways via electrospinning nanofibers of semiconducting polymer, then infiltrating with an electron acceptor. Electrospinning induces chain and crystallite alignment, resulting in enhanced light‐harvesting and charge transport. Here, the charge transport capabilities are predicted, and charge separation and dynamics are evaluated in these active layers, to assess their photovoltaic potential. Through X‐ray and electron diffraction, the fiber nanostructure is elucidated, with uniaxial elongation of the electrospinning jet aligning the polymer backbones within crystallites orthogonal to the fiber axis, and amorphous chains parallel. It is revealed that this structure forms when anisotropic crystallites, pre‐assembled in solution, become oriented along the fiber– a configuration with high charge transport potential. Competitive dissociation of excitons formed in the photoactive nanofibers is recorded, with 95%+ photoluminescence quenching upon electron acceptor introduction. Transient absorption studies reveal that silver nanoparticle addition to the fibers improves charge generation and/or lifetimes. 1 ns post‐excitation, the plasmonic architecture contains 45% more polarons, per exciton formed, than the bulk heterojunction. Therefore, enhanced exciton populations may be successfully translated into additional charge carriers.

## Graphical abstract

Nanofibrous active layers, created by electrospinning webs of ultrafine poly(3‐hexylthiophene) (P3HT) nanofibers infiltrated by [6,6]‐phenyl‐C_61_‐butyric acid methyl ester (PCBM), offer hierarchical control over molecular structure and component domains. Its photovoltaic potential is assessed by determination of the crystallite nanostructure and evaluation of competitive exciton dissociation with 95%+ quenching efficiency. Further addition of plasmonic nanoparticles may improve charge carrier generation and/or lifetimes.

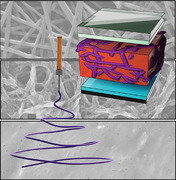

## 1 Introduction

Emerging solar technologies including organic photovoltaics (OPVs) provide intrinsic advantages over their conventional silicon counterparts. Consisting of Earth‐abundant elements, tuneable organic semiconductors provide a low‐cost, lightweight, and renewable source of energy at the point‐of‐use. Payback of the total lifetime energy requirement of commercial OPVs can be achieved in just 2–7 months^[^ ^1^, ^2^ ^]^ and are projected to hold lifetimes of 20+ years.^[^ ^2^, ^3^ ^]^ Furthermore, OPVs do not face the ecotoxicological concerns associated with competing lead perovskites^[^ ^4^ ^]^ and offer simple end‐of‐life treatment.^[^ ^5^ ^]^

The strong exciton binding energies exhibited by organic semiconductors necessitate the use of an electron donor: acceptor heterojunction to provide the driving force for charge separation. The archetypal OPV active layer, the bulk heterojunction (BHJ), maximizes this interfacial area via phase separation of a 3D donor and acceptor blend, to generate percolated, nanoscale domains. However, with the BHJ displayingpeak efficiency at <200 nm thick, it cannot harvest the complete photon flux.^[^ ^6^, ^7^ ^]^ Increasing the BHJ thickness encourages greater absorption, but to the detriment of charge extraction. State‐of‐the‐art devices lose ≈20% of potential photocurrent due to this incomplete absorption^[^ ^8^, ^9^ ^]^ with record efficiency devices reaching 80% of the Shockley‐Queisser short circuit current limit.^[^ ^9^ ^]^ Therefore, research into alternative architectures, such as nanofibrous active layers, aims to enhance light‐harvesting by a given mass of material.

**Figure 1:**
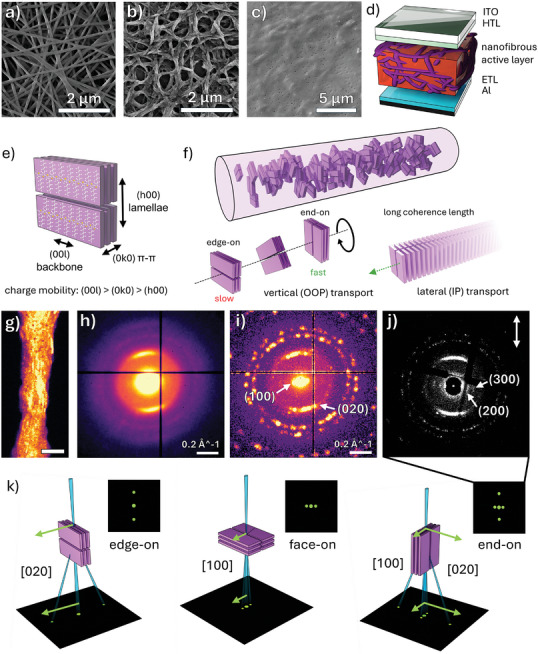
SEM micrographs of a) NP‐containing P3HT/PEO NFs, b) NP‐containing P3HT NFs after PEO removal, and c) the nanofibrous active layer following PCBM infiltration. d) Depiction of an OPV device featuring the nanofibrous active layer. Schematic of e) a simplified P3HT unit cell and f) the proposed crystallite structure within electrospun P3HT NFs, with potential mechanisms for greater lateral (in‐plane, IP) and vertical (out‐of‐plane, OOP) charge transport. g) Virtual dark field (vDF) image of a P3HT/AuNP NF (scale bar: 100 nm) generated by masking the (020) scattering region in the h) mean or i) max diffraction patterns produced by 4D‐STEM. j) Bragg vector map (BVM) of the P3HT/AuNP NF produced using a cross‐correlation protocol and corrected for beam‐shift, elliptical distortion, and the real‐to‐diffraction space rotation. Arrow denotes the real‐space fiber orientation. k) Expected electron diffraction patterns generated by the edge‐on, face‐on, and end‐on P3HT crystallite configurations.

Such active layers are produced as depicted in **Figure** **1**a–d, by electrospinning a nanofiber (NF) mat of an electron donor polymer, e.g. poly(3‐hexylthiophene) (P3HT), and subsequently infiltrating the interstitial space with an acceptor^[^ ^10^, ^11^, ^12^ ^]^ or BHJ blend.^[^ ^13^, ^14^ ^]^ The infiltration method for incorporating electrospun material into OPV active layers was initially developed by Bedford et al.,^[^ ^13^ ^]^ adapted to blade‐coating by Pierini et al.,^[^ ^15^ ^]^ and altered to deposit acceptor molecules from orthogonal solvents by Kim et al., as in this work.^[^ ^10^, ^11^ ^]^

This architecture provides opportunities to engineer the active layer structure beyond the limits of the BHJ, at the molecular, crystallite, and domain scales. Electrospinning is a scalable method of producing continuous, nanoscale fibers through the extrusion of a jet from a polymer solution by strong electric fields. The shear and elongational forces experienced by the fiber commonly induce polymer chain extension and alignment at the molecular level. Upon fiber collection, polymer backbones are oriented within the substrate plane, optimizing overlap between the π→π* transition dipole moment and the electric field vector of light^[^ ^13^, ^14^ ^]^ and producing a red‐shift, delivering greater optical absorption.^[^ ^13^, ^15^, ^16^ ^]^ Chain and crystallite alignment may also lead to increased charge mobility. When appropriately implemented, nanofibrous active layers have improved device efficiencies relative to their thin‐film equivalents.^[^ ^11^, ^13^, ^14^, ^15^ ^]^

This active layer retains the benefits of a bulk‐like interface with bi‐continuity of the donor and acceptor domains, whilst offering additional control at the domain level. Design of the donor NFs allows tuning of the domain size,^[^ ^10^, ^11^ ^]^ the intermixed region,^[^ ^17^ ^]^ or the distribution/grading of each component. These architectures could also deliver effective nanoscale phase separation in donor: acceptor systems which cannot be processed from blend solutions due to excessive miscibility or immiscibility.^[^ ^18^, ^19^ ^]^

Although sequential deposition requires the use of orthogonal solvents, it would be possible to select the delivery of key additives, such as plasmonic nanoparticles (NPs), to specific regions. Resultantly, in the plasmonic, nanofibrous active layer presented here, the dominant photoactive component can be preferentially located in the region of greatest electric field enhancement, at the NP surface.^[^ ^20^ ^]^ It is important to note that NPs are not primarily (or exclusively) introduced into electrospun fibers to harness plasmonic enhancement. Instead, our previous research revealed that NP addition to P3HT NFs produced thinner diameter fibers than previously accessible, generating a greater degree of polymer alignment which enhanced photon absorption by increasing the population of polymer chains oriented in‐plane. Alongside the Local Surface Plasmon Resonance (LSPR) effect, NP‐containing P3HT NFs showed the absorption of 58% more light than thin films.^[^ ^16^ ^]^

The present work seeks to evaluate the ultimate potential of the plasmonic, nanofibrous active layer based upon the conventional donor and acceptor, P3HT, and [6,6]‐phenyl‐C_61_‐butyric acid methyl ester (PCBM). By investigating the efficiency of individual photovoltaic processes—namely exciton dissociation and the kinetics of resultant charges—whilst also forecasting charge transport properties through structural studies, this study aims to predict the performance of an optimized nanofibrous OPV device. An inefficiency in any one of these steps could impose a bottleneck on the entire system, highlighting the importance of this stepwise investigation, which has not yet been conducted upon electrospun active layers. The majority of existing research has focused on the overall performance of complete devices featuring electrospun fibers, leaving a significant gap in understanding the efficacy of fundamental processes and process‐structure‐property relationships.

The classical P3HT:PCBM donor:acceptor system serves as an ideal prototypal platform for novel architectures, enabling the study of the effects of fiber processing on key photovoltaic steps. The photo‐physics and morphology of this blend are well‐studied, making it highly attractive for preliminary studies, despite its comparatively lower photovoltaic efficiency relative to contemporary materials. The incorporation of an auxiliary polymer to control electrospinning facilitates the straightforward transfer of this architecture to higher‐efficiency systems, without significant changes to processing. This approach ensures that the insights gained here can be directly applied to state‐of‐the‐art OPVs, such as push‐pull type polymers and Y‐series acceptors.

Furthermore, these advancements in non‐fullerene acceptors and push‐pull polymer donors have led to laboratory OPV devices with efficiencies exceeding 19%.^[^ ^21^ ^]^ Arguably, this satisfies commercialization barriers,^[^ ^22^, ^23^ ^]^ and therefore, the primary research focus is shifting from driving efficiencies higher to replicating these achievements with materials that are cheaper, chemically simpler, and more air‐stable.^[^ ^5^, ^23^ ^]^ These qualities are better presented by the classical P3HT:PCBM system versus those used in current record devices.^[^ ^24^, ^25^, ^26^ ^]^

To assess charge transport in P3HT fibers, we investigate how the electrospinning process dictates the molecular structure of the photoactive material. X‐ray and 4D‐Scanning Transmission Electron Microscopy (4D‐STEM) diffraction is used to elucidate the crystallite orientation, anisotropic dimensions, and distribution within individual P3HT NFs. To our knowledge, this represents the first published application of 4D‐STEM to map the crystalline phases within electrospun nanofibers. We find that polymer backbones within the crystallites are oriented orthogonally in the fiber, which holds the possibility for enhancing lateral (IP, along the fiber axis) and vertical (OOP, across the fiber) charge transport (Figure 1e,f).

Excitons must be dissociated for good photovoltaic performance. Historically, charge separation in NF‐based heterojunctions has been ineffectual, as fiber diameters were often magnitudes larger than the exciton diffusion length due to the difficulty in spinning conjugated polymers.^[^ ^27^ ^]^ Hence, it is common to co‐electrospin with an auxiliary polymer to generate the entanglement to reliably spin homogenous fibers. In combination with polar co‐solvent addition,^[^ ^10^ ^]^ or the introduction of plasmonic metal NPs to spinning solutions, ultra‐thin P3HT NFs have been produced with diameters of just 55 nm.^[^ ^16^ ^]^ We demonstrate that these diameter reduction strategies ameliorate exciton recombination issues which hamper the performance of thicker fibers. Competitive levels of charge carrier generation are revealed using photoluminescence (PL) and femtosecond‐Transient Absorption Spectroscopy (fs‐TAS), to probe the dynamics of photoinduced species over four temporal decades. Here, the direct evidence of charge carrier formation acquired via fs‐TAS not only substantiates but also conclusively validates the inferences from PL quenching used in prior research. Active layers comprised of fibers with diameters a magnitude larger than the exciton diffusion length exceeded predictions, providing near‐complete PL quenching, likely due to PCBM diffusion into NFs.

Whilst plasmonic NPs reproducibly increase initial exciton populations, their influence on device performance remains controversial,^[^ ^28^, ^29^, ^30^ ^]^ due to recombination mechanisms introduced following NP addition.^[^ ^20^ ^]^ Contrary to prior reports, here, fs‐TAS provides evidence of slower exciton/polaron signal relaxation in nanofibrous active layers, indicative of more effective charge separation and longer lifetimes. This finding challenges long‐standing concerns and demonstrates that when integrated appropriately, plasmonic nanoparticles can be beneficial rather than parasitic.

## 2 Results and Discussion

### 2.1 Elucidation of Internal Crystallite Nanostructure via Electron and X‐ray Diffraction

Pristine and NP‐containing P3HT NFs were prepared directly onto indium tin oxide (ITO) coated glass substrates using the electrospinning protocol outlined by Schofield et al.^[^ ^16^ ^]^ P3HT and other conjugated polymers produce low entanglement in solution, and therefore poly(ethylene oxide) (PEO) is added to increase solution viscosity. During electrospinning, PEO entanglement resists the elongational forces generated by the electric field upon the charged jet, impeding fiber break‐up or beading, and promoting uniform/homogeneous morphologies.^[^ ^31^, ^32^ ^]^ The addition of AuNPs (capped with oleylamine) or AgNPs (produced in situ following the introduction of AgNO_3_) reduces the viscosity and increases the conductivity of PEO solutions over time,^[^ ^33^ ^]^ which reduces the diameter of fibers produced from solutions aged for longer before spinning.^[^ ^16^ ^]^

Thus, P3HT/PEO‐based NFs were first prepared from blend solutions, aged for 96 h (Figure 1a), and P3HT NFs were then obtained by submerging in isopropanol (IPA), heated above the glass transition temperature of PEO (Figure 1b). In our previous work, thermogravimetric analysis was used to demonstrate that this procedure was up to 98.5% effective at selectively removing PEO.^[^ ^16^ ^]^ The resultant NP‐containing fibers measure ≈55 nm in depth (diameter normal to the substrate) by 100 nm wide (in the plane of the substrate). Pristine NFs are larger, at 100 nm deep by 330 nm wide.^[^ ^16^ ^]^

Figure 1e depicts a simplified P3HT crystal lattice, defining the directional terminology and (hkl) axes used in the following section. Hole mobility in P3HT is fastest along the backbone, then in the π–π direction, and slowest in the lamellae direction,^[^ ^34^ ^]^ while longer crystallite coherence lengths reduce resistance to charge transport. As OOP charge transport is vital for carrier collection in OPVs,^[^ ^35^ ^]^ it is essential to understand the orientation of crystallites within the nanofibrous active layers wrt the substrate. To investigate the influence of electrospinning on the crystallite orientation, we utilize 4D‐STEM and 2D Grazing Incidence X‐ray Diffraction (2D GI‐XRD). These studies reveal a preferential alignment of P3HT crystallites within the electrospun fibers, more specifically, that presented in Figure 1f.

Direct observation of crystallites within beam‐sensitive organic materials is challenging,^[^ ^36^, ^37^, ^38^, ^39^ ^]^ due to rapid loss of polymer crystallinity via radiolysis upon electron exposure.^[^ ^40^ ^]^ In 4D‐STEM nanobeam diffraction, an electron beam, ≈2 nm in diameter, is raster scanned across a specimen and a 2D diffraction pattern is recorded at each beam position. At non‐cryogenic conditions, low electron doses (<6 e^−^ Å^−2^) in combination with a blind fiber selection strategy, were sufficient to reduce beam damage and enable the collection of clear diffraction from P3HT/AuNP NFs, shown in the virtual dark field (vDF) image in Figure 1g. The mean and maximum diffraction patterns produced by this NF are displayed in Figure 1h,i. Bragg spots, originating from P3HT (020) π–π and (100) lamellar stacking, are visible at 0.525 Å^−1^ (0.38 nm) and ≈0.131 Å^−1^ (≈1.53 nm), respectively. A representative selection of diffraction patterns is given in Figure S1 (Supporting Information).

The mean diffraction pattern in Figure 1h is dominated by the prominent P3HT (020) diffraction. However, in the maximum diffraction pattern in Figure 1i, each pixel is assigned the greatest intensity recorded across all probe positions. Thereby, Bragg spots which are present at very few electron probe positions can be visualized. In this scheme, diffraction by the (111), (200), (220), (311), and (222) planes of the AuNPs, dispersed within the polymer matrix, is better defined (labeled in Figure S2, Supporting Information) and P3HT (100) stacking is observed beside the central disc.

Perfect P3HT crystals produce a series of well‐defined Bragg spots,^[^ ^41^, ^42^ ^]^ however, the diffuse (020) and (100) scattering recorded here, indicates that the fibers are polycrystalline—consistent with select‐area electron diffraction (SAED) analysis on P3HT:PCBM fibers, electrospun by Bedford et al.^[^ ^13^ ^]^ Although, conversely to that report, we find that the (020) and (100) scattering is localized to azimuthal ranges at 90° β to each other. This reveals that a preferential orientation is generated within the fibers, with the observed scattering behavior consistent with end‐on crystallites, or a combination of two or more configurations, as conveyed in Figure 1k. P3HT configurations are typically denoted wrt the substrate, or the plane of the TEM grid in this case.

The vDF in Figure 1g is generated by placing a virtual annular aperture in the (020) scattering region of the raw diffraction patterns (shown in Figure S3b, Supporting Information) and reveals the anisotropic shape of these features, which appear as a series of long striations along the fiber axis. P3HT lamellar features or the AuNPs may also be visualized analogously, see Figures S3 and S4 (Supporting Information). Using a cross‐correlation template matching technique (parameter tuning in Figure S5, Supporting Information), the position of all Bragg reflections in the 4D dataset are identified. This protocol detects the Bragg peaks generated by crystalline regions by finding the cross‐correlation maxima, or features of closest similarity, to a template of the vacuum probe.^[^ ^37^, ^43^, ^44^ ^]^ This reduces the background scattering intensity in the Bragg vector map (BVM) as contributions from amorphous scattering and noise do not match the structure of the template, limiting false detection.^[^ ^43^ ^]^ Therefore, in the BVM presented in Figure 1j, higher‐order P3HT (200) and (300) spacings are also resolved. Following the rotation calibration, it is revealed that the [0k0] crystallite axis is oriented along the fiber, whilst the [h00] axis is found across the fiber.

**Figure 2:**
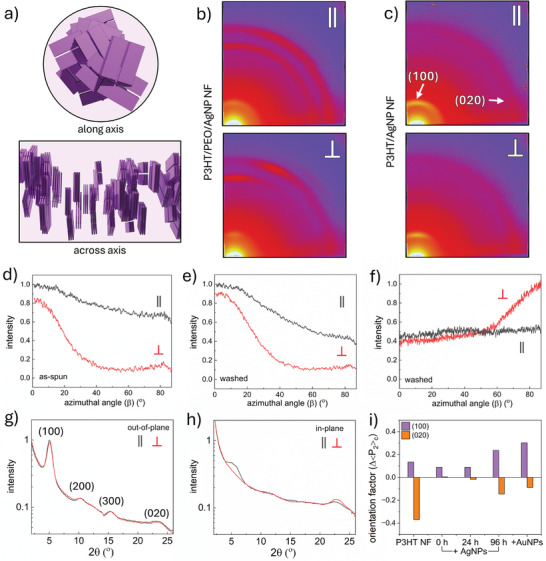
a) Schematic presenting the along and across‐axis view of the proposed crystallite orientation in electrospun P3HT NFs. b,c) 2D GI‐XRD detector images produced by aligned nanowebs of b) as‐spun P3HT/PEO/AgNP NFs and c) washed P3HT/AgNP NFs with the X‐ray beam incident parallel (ǁ) and perpendicular (⊥) to the fiber axes. Scattering characteristics are largely isotropic with parallel radiation but reveal a preferential orientation when perpendicular. d–f) Azimuthal line‐cuts from the OOP meridian (0 °β) to IP axis (87 °β) of d,e) (100) scattering of d) as‐spun and e) washed NFs. f) Azimuthal trace of (020) scattering by washed NFs. g) OOP and h) IP 1D profiles of washed P3HT/AgNP NFs. i) The extent of crystallite alignment in varied diameter P3HT NFs determined from the difference in Hermans’ orientation factor with parallel versus perpendicular radiation.

To elucidate the specific crystallite configurations, 2D GI‐XRD is employed in **Figure** **2**, wherein the X‐ray radiation is incident parallel and perpendicular to the fiber axes (see Figures S6–S10, Supporting Information), with the parallel view enabling investigation of the “radial” preference of crystallites within the fiber. This is achieved using a rotating collector to generate fiber‐to‐fiber alignment in a common direction. In Figure 2a, the cross–sectional and side view of the proposed fiber nanostructure from Figure 1f is presented.

As the fiber jet twists during the flight from the spinneret to the collector, one may not expect a preferred crystallite orientation wrt the fiber radius to exist. Indeed, with the parallel (ǁ) fiber‐to‐beam view, the 2D detector images in Figure 2b contain largely isotropic Debye‐Scherrer rings from P3HT (100) scattering, at 5–5.5° 2θ, and PEO scattering at 19° and 23° 2θ, predominantly arising from (112) and (120) planes, respectively. The broader intensity of IP scattering is discussed in Figure S7 (Supporting Information). Conveyed in Figure 2d, the azimuthal traces, extracted from the OOP meridian, 0° β, to the IP axis, 87° β, indicate a continuum of (100) planes oriented from approximately parallel to the substrate (scattering OOP) to perpendicular (scattering IP). The IP (100) scattering intensity reaches 70% of its OOP maximum, and therefore, there is some preference for the edge‐on orientation overall. When the fiber is collected, the residual solvent could provide polymer chain lability to enrich the relative proportion of edge‐on crystallites, as it is thermodynamically favorable for alkyl side‐chains to be located at the substrate‐fiber interface. Thermal treatment during PEO removal further enhances the edge‐on preference, and in response, the IP (100) intensity drops to 40% versus OOP (Figure 2e). This is accelerated by NF slumping during washing, which increases the substrate‐fiber interfacial area.

It is clear from the along‐axis (parallel) view in Figure 2a, that P3HT (020) planes should not produce scattering with this X‐ray orientation, however, imperfect crystallite alignment and macroscopic fiber‐to‐fiber alignment deliver a weak, isotropic (020) ring. This is observed in Figure 2c at 22–23° 2θ after PEO dissolution.

When X‐rays are perpendicular (⊥) to the fiber, equivalent diffraction characteristics to that presented in Figure 1j are generated. This indicates that (100) planes that satisfy the Bragg condition are oriented parallel to the substrate, producing a strong preference for OOP scattering (see ⊥ in Figure 2b–e). (020) planes are perpendicular to the substrate, producing the opposite IP scattering preference (see ⊥ in Figure 2c,f). Randomly‐oriented fiber mats produce averaged behavior of each X‐ray orientation, Figure S11 (Supporting Information).

These results corroborate a regime where crystallites, with the [0k0] axis oriented along the fiber length, are rotated about the fiber axis, reducing the radial preference and producing a continuum of crystallite orientations from edge‐on to end‐on wrt the substrate. Inspection of the extracted 1D OOP (0‐ 10 °β) and IP (77‐ 87 °β) profiles in Figure 2g,h, relay a similar story, with almost identical OOP scattering regardless of the orientation. Meanwhile, IP (100) and (020) scattering are higher under parallel and perpendicular fiber‐to‐beam conditions, respectively.

Hermans’ orientation factors (<*P*_2_>) can determine the extent of alignment from the azimuthal traces (discussion following Figure S12 and Table S1, Supporting Information). Δ<*P*_2_>, or the level of along‐fiber alignment, is estimated from the difference between the factors calculated with parallel and perpendicular X‐ray illumination. In Figure 2i, Δ<*P*_2_> of NFs spun after the initial AgNO_3_ addition drops to near‐zero. Yet as the solution is aged before fiber spinning, Δ<*P*_2_> increases alongside the diameter reduction (see also Figures S13–S16, Supporting Information). The ultra‐thin NP‐containing P3HT NFs (aged for 96 h) possess greater alignment of (100) planes parallel to the substrate than their thicker, pristine counterparts. However, the pristine NFs retain a greater perpendicular alignment wrt (020) planes. Therefore, a systematic relationship between the degree of crystallite alignment and diameter is not found.

Crystallites are frequently reported to form with the polymer backbone oriented along the fiber^[^ ^13^, ^45^ ^]^ as stretching forces extend polymer chains in the direction of elongation during the early stages of electrospinning. As the fiber dries, the chains template crystallization, with the [00l] axis orientation becoming kinetically trapped along the fiber. This nanostructure would produce greater OOP (100) and (020) scattering in the perpendicular X‐ray regime.^[^ ^13^, ^45^, ^46^, ^47^, ^48^ ^]^

With the perpendicular fiber‐to‐beam orientation, PEO crystallites in the as‐spun fibers present scattering behavior akin to studies wherein the backbone is oriented along the fiber axis with no radial preference‐ supported by the isotropic scattering character with the parallel X‐ray beam.^[^ ^49^, ^50^ ^]^ Furthermore, in our previous study on these P3HT NFs, polarized absorption spectroscopy revealed an overall preference for the polymer chains to be oriented along the fiber backbone.^[^ ^16^ ^]^ Therefore, it was intriguing that the polymer chains in the crystallites are found to be orthogonal to the fiber. Although this is precedented, the origin of its formation has remained elusive.^[^ ^51^, ^52^ ^]^ The aforementioned polarized absorption is a sum of amorphous and crystalline polymer contributions. In combination, these analyses reveal that our electrospun NFs present a unique internal structure; one wherein polymer chains in amorphous domains are oriented along the fiber backbone, whilst polymer in crystalline regions possesses an orthogonal orientation.

**Figure 3:**
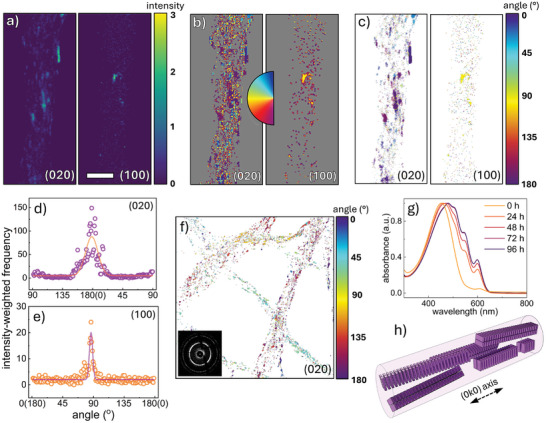
a) Intensity and b) angle/orientation map of (020) and (010) Bragg scattering at each probe position. Color in (b) is assigned based on the angle between the Bragg vector and the vertical axis. c) Combined intensity‐weighted orientation map with color governed by the angle, whilst intensity dictates opacity. d,e) 2D histogram of the d) (020) and e) (100) Bragg vector angles extracted from (c). The continuous line represents a gaussian curve fit. f) Intensity‐weighted angle map of a P3HT nanoweb featuring several fibers (inset: BVM of the web). g) UV–Vis absorption spectra of the spinning solution at different solution aging times, revealing crystallite self‐assembly prior to electrospinning. h) Idealized structural schematic of crystallite anisotropy and orientation within electrospun P3HT NFs, crystallites not to scale.

To reveal the mechanism for this nanostructure's formation, vDF images are constructed from the 4D‐STEM BVM from the (100) and (020) scattering regions. Probe positions are assigned values according to the maximum recorded intensity of (100) and (020) diffraction, and the angle between a line drawn from the central disc to the Bragg peak, and the *y*‐axis. The corresponding maps are displayed in **Figure** **3**a,b. After Bragg detection, the crystalline features in Figure 3a,b are more distinct than Figure 1g and Figure S3 (Supporting Information), and now reveal the spatial distribution and anisotropic shape of the crystallites– with π–π coherence lengths imaged up to 80 nm (≈10 nm wide).

Combining the intensity and angular position of the Bragg peaks generates an intensity‐weighted angle map presented in Figure 3c, using intensity to assign opacity to each pixel. (100) scattering is detected at fewer probe positions than (020), as (020) planes will satisfy the scattering condition throughout the rotation of a P3HT crystallite about its [0k0] axis. Conversely, a 2° tilt from the [00l] axis, attenuates (100) scattering (Figure S17, Supporting Information). Histograms of intensity and Bragg peak angle, are shown in Figure 3d,e. The intensity‐weighted angle map (Figure 3c) demonstrates the high degree of common orientation imparted by the electrospinning process within a single fiber. The <*P*_2_> factor of the (020) map in Figure 3d is 0.639 (0.593 ± 0.116, *n* = 7) rising to 0.743 (0.636 ± 0.110) when weighted for intensity; greater alignment is determined than with X‐rays due to the removal of amorphous contribution under the Bragg detection method. The intensity‐weighted (020) map of a nanoweb containing several randomly‐oriented fibers is displayed in Figure 3f. The inset BVM presents isotropic behavior across the full field of view, however 4D‐STEM reveals the alignment within each fiber.

In Figure 3d, a sub‐peak is present 90° β to the major (020) peak, attributed to either (020) or (002) scattering, which possess similar d‐spacings. If ascribed to (002), this indicates that pre‐aggregation produces substantial order in the backbone direction, not observed in thin films or previously reported electrospun P3HT fibers. If attributed to (020), this would be ascribed to crystallites formed during electrospinning, templated by dissolved polymer chains that are aligned parallel to the fiber prior to crystallization.

The development of vibronic peaks over time in Figure 3g, co‐existing with the π→π* transition of well‐dissolved P3HT at 451 nm, indicates that pre‐aggregation of crystallites occurs in solution following N,N'‐dimethylformamide (DMF) addition and aging. In fact, non‐solvent addition or slow cooling are routine methods used to produce self‐assembled crystals.^[^ ^41^, ^42^ ^]^ These crystals are often anisotropic, with P3HT growing preferentially by π–π stacking, and are therefore longest in this direction, as in Figure 3a–c.^[^ ^38^, ^53^, ^54^, ^55^ ^]^ Therein, we reveal that the formation mechanism for crystalline domains containing orthogonally‐oriented chains is the very same driving force that aligns the polymer in amorphous regions along the axis‐ uniaxial elongation. Dissolved polymer chains are extended along the fiber axis as the molecule is longest in the backbone direction. However, once assembled into nanocrystals, the longer aspect is in the (0k0) direction, orthogonal to the (00l) backbone. Thereby, elongation delivers the opposite backbone orientation.

These findings suggest that polymer crystallinity is dominated by crystallites formed by pre‐aggregation before spinning, rather than during the spinning process. Both high and low crystallinity in electrospun fibers versus films have been frequently observed.^[^ ^10^, ^52^ ^]^ Here, it is evident that rapid evaporation of the volatile solvent, chloroform, does not allow for significant additional crystallization to occur during electrospinning. X‐ray studies of the P3HT/AgNP NFs prepared from lesser‐aged solutions (0‐ 24 h) appear to indicate a lower extent of crystallinity, as the P3HT crystallites pre‐assemble over time (see Figures S13 and S14, Supporting Information). This finding is significant as it reveals that pre‐aggregation via solution aging or non‐solvent addition provides a means to tune the extent of crystallinity within electrospun organic semiconductor fibers toward charge mobility optimization. As crystallites may be aligned after their formation via this approach, it provides an advantage over annealing, as thermal treatment reportedly degrades the electrospinning‐induced alignment and charge mobility, despite increasing crystallinity.^[^ ^56^, ^57^ ^]^ As crystalline regions are not percolated within P3HT thin films, transport through the often rate‐limiting amorphous regions is required.^[^ ^58^, ^59^ ^]^ However, crystallites can be considered electrically continuous if a tie‐chain, with a conjugation length comparable to the inter‐crystallite distance, spans the gap.^[^ ^60^ ^]^ The alignment of amorphous chains along the NF could improve crystallite interconnectivity by extending tie‐chains and their conjugation lengths, thus, offsetting the effect of lower crystallinity.

The axial alignment of uninterrupted, long‐range π–π stacking along the fiber length should be advantageous for efficient in‐plane charge transport. This direction of transport is highly desirable within devices such as organic field‐effect transistors. Additionally, the range/continuum of edge‐on to end‐on configurations found in the NFs holds the potential for enhanced OOP (vertical) mobility, owing to the increased prevalence of the end‐on configuration versus thin films, directing transport along the polymer backbone.

Edge‐on epitaxy is well reported for P3HT thin films produced by spin‐coating slow‐drying solutions, Figure S9b (Supporting Information).^[^ ^61^ ^]^ However, the end‐on or face‐on orientations are theoretically preferred for OOP mobility.^[^ ^61^, ^62^ ^]^ Face‐on (or isotropic) orientations can be produced in rapidly‐drying films due to kinetic trapping, but the crystallites are of lower quality and offer inferior performance,^[^ ^63^, ^64^ ^]^ whilst the end‐on configuration is challenging to achieve.^[^ ^65^ ^]^ In the BHJ blend, electron acceptor introduction disrupts epitaxy/crystallization (Figure S18a, Supporting Information), and so, thermal^[^ ^66^ ^]^ or solvent^[^ ^67^ ^]^ annealing is used ubiquitously, which re‐generates edge‐on epitaxy. Hence, edge‐on P3HT BHJs provide sub‐optimal OOP mobility due to the insulating alkyl side‐chains interdigitated in the lamellae direction. This disruption is not observed when electrospun P3HT NFs are infiltrated with PBCM to produce a nanofibrous active layer, Figure S18b (Supporting Information). Therefore, the crystallite structure of the fibers in this architecture shows promise for enhancing both IP and OOP charge transport, due to long persistence lengths and a greater end‐on population wrt the substrate.

### 2.2 Competitive Exciton Separation Within Nanofibrous Active Layers

Noble metal NP addition to P3HT NFs produces broadband light‐harvesting enhancements, with LSPR and in‐plane polymer alignment delivering integrated absorptance enhancements of 48% (AgNPs) and 58% (AuNPs) versus films.^[^ ^16^ ^]^ However, to lead to an improvement in photovoltaic performance, the exciton populations formed in the NFs must reach the donor: acceptor heterojunction before quenching, for charge separation. Therefore, exciton dissociation within the active layer is evaluated by PL quenching and fs‐TAS experiments.

Pristine and NP‐containing P3HT NF webs were electrospun onto ITO for 120 s before the interstitial space was backfilled with PCBM via spin‐coating from dichloromethane (DCM). The SEM micrograph in Figure 1c confirms successful fiber encapsulation and the formation of a nanofibrous heterojunction with a thickness of 200‐ 300 nm (Figure S19a, Supporting Information). Atomic Force Microscopy (AFM) height micrographs reveal that the fibrous active layer possesses a root mean square (RMS) surface roughness of 1.3 ± 0.2 nm, exceeding that of the BHJ (0.5 ± 0.1 nm). (Figure S19b,c, Supporting Information) This finding/value is consistent with previous works wherein electrospun mats have been infiltrated with BHJ blends.^[^ ^13^, ^15^ ^]^ This increased roughness can be advantageous for charge extraction.^[^ ^68^ ^]^ However, the pinholes in the PCBM film should be alleviated to avoid shunting concerns in final devices. Energy‐dispersive X‐ray (EDX) studies were conducted on a low‐density P3HT/AuNP NF: PCBM active layer to confirm that the fiber morphology is retained after exposure to the orthogonal solvent (Figure S20, Supporting Information). Long‐range NP pathways are maintained within the active layer and line‐scans (S Kα1) indicate that sulfur‐containing P3HT is localized to these regions. AFM topology (Figure S21, Supporting Information) of the nanoweb before and after spin‐coating pure DCM reveals that infiltration reduces the web depth from ≈500 to ≈140 nm. This NF densification helps prevent the formation of excessively large acceptor domains, dictated by the interstitial space.

**Figure 4:**
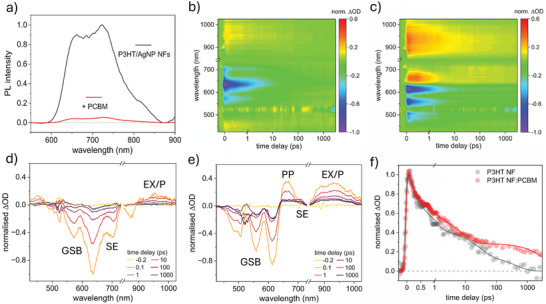
a) PL quenching efficiency upon PCBM infiltration of P3HT/AgNP NFs to generate a plasmonic, nanofibrous active layer. Spectra were normalized to the PL intensity of the NF (0–1) transition. b,c) fs‐TAS false color heat maps and d,e) differential optical spectra at selected time delays of (b,d) P3HT NFs and (c,e) P3HT NF:PCBM, normalized to the greatest GSB intensity (−1.0 ΔOD) after excitation with a 520 nm pump pulse. f) Transient relaxation of the exciton/polaron (EX/P) signal before and after PCBM infiltration, monitored at 950 nm probe wavelength and normalized to the maximum ΔOD after photoexcitation.

PL quenching efficiency can be used to predict eventual performance, with higher efficiency coinciding with greater quenching probability.^[^ ^69^, ^70^ ^]^ Prior to PCBM introduction in **Figure** **4**a, there is significant radiative quenching of excitons formed within the NFs. Following infiltration, >95% of PL emission is attenuated, signifying successful electron transfer to the fullerene region. Equivalent analysis of a P3HT:PCBM BHJ shows that 99.85% of PL emission measured from a P3HT thin‐film is quenched upon PCBM blending (Figure S22a, Supporting Information). Kim et al. previously demonstrated that PL quenching was intimately linked to diameter, as thinner fibers deliver shorter exciton diffusion pathways.^[^ ^10^, ^11^ ^]^ However, in Figure S22 (Supporting Information), the pristine P3HT NF:PCBM active layer outperformed expectations, despite the fiber diameters being a magnitude larger than typical P3HT diffusion lengths (≈10 nm). This is likely due to significant PCBM diffusion into the P3HT NFs, despite the use of an orthogonal solvent,^[^ ^71^ ^]^ which reduces the distance to the interface. Alternatively, there exists evidence of greater exciton diffusion lengths within electrospun donor polymer fibers.^[^ ^72^ ^]^ Recently, Sneyd et al. reported that long π–π coherence lengths in self‐assembled crystallites, which we have postulated exist within these NFs, offer diffusion lengths up to 300 nm.^[^ ^38^ ^]^ Such performance indicates that ideal NFs for OPVs can exceed a diameter of twice the exciton diffusion length, yet ultra‐thin diameters remain advantageous for delivering a high chain and crystallite alignment.

We note that the NF PL possesses an enhanced (0‐0) vibronic transition at 660 nm, relative to (0–1) at 720 nm, compared to the thin‐film, rising from 0.73 (film) to 0.90 (NFs). This is indicative of reduced H‐aggregate character versus thin films with increasing intrachain interactions.^[^ ^73^, ^74^, ^75^ ^]^ The (0–0) transition is red‐shifted by 8 nm upon electrospinning, signifying longer polymer conjugation lengths.

To produce direct evidence that PL quenching results in the generation of free charge carriers, fs‐TAS studies were conducted on the NFs before and after PCBM infiltration in the temporal range of 40 fs to 3 ns. In Figure 4b, the TAS false color heat maps of the P3HT NFs are given, from which differential optical density (ΔOD) spectra are extracted at select time delays, presented in Figure 4d. Negative ΔOD signals arise from ground state bleaching (GSB) upon molecular photoexcitation by the 520 nm pump pulse. The peaks at ≈635 nm and 570 nm relate to the (0–0) and (0–1) P3HT vibronic transitions. In the corresponding plots after PCBM backfill in Figure 4c,e, these peaks blue‐shift to ≈615 nm (0–0) and 560 nm (0–1). The (0–2) transition is also visible at ≈495 nm. Blue‐shift may arise due to attenuation of the (0–0) transition as PCBM disrupts packing and conjugation lengths, and the evolution of a photoinduced absorption (PIA) signal, centered at 660 nm.

The positive peak at 660 nm has been most convincingly assigned to polaron pairs (PP, an intermediate charge‐transfer state)^[^ ^76^, ^77^ ^]^ but also to P3HT polarons^[^ ^78^ ^]^ (a charge carrier plus the electronic distortion) in crystalline domains.^[^ ^79^ ^]^ Distinctly, without PCBM, this peak is not present (Figure 4b,d). Instead, a negative ΔOD signal is observed at ≈720 nm, attributed to the stimulated emission (SE) of P3HT excitons^[^ ^76^ ^]^ which relaxes within 100 ps (Figure S23, Supporting Information).^[^ ^28^ ^]^ Weak SE is found in the TAS spectra of the P3HT NF:PCBM architecture as an inflection upon the more intense PP signal.

Singlet P3HT excitons (EX) are typically observed as a broad peak at 1200 nm (spanning 800–1400 nm).^[^ ^38^, ^80^ ^]^ In this study, the initial PIA at >850 nm captures the shoulder of this exciton peak. In the presence of PCBM, excitons are rapidly dissociated within 10s of ps,^[^ ^28^ ^]^ leaving a relatively long‐lived polaron signal (on the order of ns) centered at ≈950–1000 nm.^[^ ^73^ ^]^ As polarons evolve from exciton separation, signal relaxation at 950 nm probe wavelength is convolved. However, the signal immediately after photo‐excitation is representative of the exciton population, and after ≈1 ns, beyond the temporal window of exciton dissociation, the residual signal at 950 nm is attributed to successfully separated polarons. This time delay was selected due to its use by Wu et al. to investigate nanoparticle‐associated recombination in P3HT:PCBM BHJs.^[^ ^28^ ^]^

Kinetics were fitted using a tri‐component exponential decay function and the polaron population 1 ns after the pump, relative to the initial excitation population, was estimated using absorption coefficients (*ε*) proposed by Okhita et al. at a 950 nm probe wavelength (details in Figure S24, Supporting Information discussion).^[^ ^76^ ^]^ Standard errors for the decay profiles at this probe wavelength are also provided in Figure S24e (Supporting Information).

In Figure 4f, the normalized 950 nm signal decays slower in the infiltrated active layer versus pristine NFs, indicating the formation of free charges. At 1 ns, the polaron signal in the NFs is just 0.8% wrt exciton generation (**Table** **1**). Conversely, polaron concentration in the nanofibrous active layer is 11.6% wrt generated excitons, a 15‐fold increase. This figure represents polarons persisting for at least 1 ns, not the total polaron generation rate. Poor polaron formation in the absence of PCBM arises as self‐dissociation in P3HT is uncommon, only enabled by a small driving force at crystalline/amorphous region interfaces,^[^ ^81^, ^82^ ^]^ or hot excitons^[^ ^76^ ^]^ (0.7 eV above the bandgap^[^ ^83^ ^]^), not generated by 520 nm excitation.^[^ ^38^ ^]^

**Table 1:** Residual signal at 950 nm after 1 ns versus initial intensity after photoexcitation and estimated polaron (P) populations using coefficients determined by Okhita et al. The relative polaron population and absolute population when compounded with absorptance enhancement reported by Schofield et al.^[^ ^16^ ^]^ Error is propagated from the standard error given for ΔOD at 1 ns (Details in Figure S24, Supporting Information).

Active Layer	ΔOD (Std. Err.) [1ns/initial]	P population at 1 ns [EX^−1^] [%]	Relative P enhancement [%]	Compounded P enhancement [%]
P3HT:PCBM BHJ	0.254 ± 0.011	12.89 ± 0.56	–	–
P3HT NF (w/o PCBM)	0.016 ± 0.022	0.80 ± 1.12	−93.8 ± 8.7	−92.9 ± 9.92
P3HT NF:PCBM	0.228 ± 0.010	11.57 ± 0.51	−10.2 ± 5.6	+2.3 ± 6.3
P3HT/AgNP NF:PCBM	0.370 ± 0.019	18.78 ± 0.96	+45.7 ± 9.8	+115.6 ± 14.5
P3HT/AuNP NF:PCBM	0.230 ± 0.011	11.68 ± 0.51	−9.4 ± 5.6	+43.2 ± 8.9

In Table S2 (Supporting Information), the fitted time constants and component amplitudes are provided. Previously, the first two components have been ascribed to excitons in intermixed regions (*A*_1_, *τ*_1_) and crystalline domains (*A*_2_, *τ*_2_).^[^ ^84^ ^]^ These time constants decrease upon PCBM introduction, indicative of rapid exciton dissociation. The amplitude of the third component, ascribed to polarons,^[^ ^84^ ^]^ is proportional to the total polaron population, which doubles from 12.5% to 25.5% with acceptor backfill. Furthermore, τ_3_ increases by an order of magnitude, to >3 ns. Combined, PL and fs‐TAS provide clear evidence that 95‐ 97% of excitons produced in nanofibrous active layers are quenched at the acceptor, and this process leads to the generation of free charge carriers – thus, concluding that these electrospun NFs are of a suitable diameter for OPV implementation.

### 2.3 Influence of Plasmonic Nanoparticles on Polaron Dynamics

**Figure 5:**
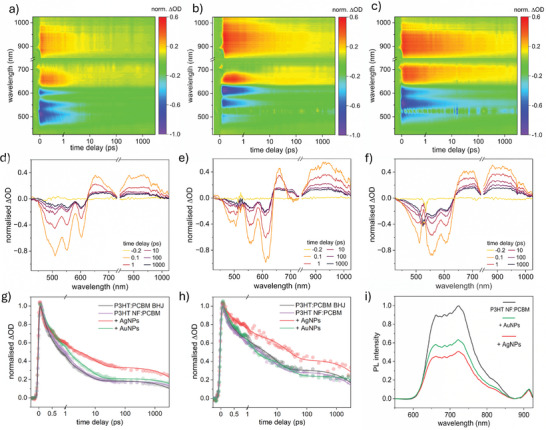
a–c) TAS false color heat maps and (d–f) differential optical spectra at key time delays of (a, d) P3HT: PCBM BHJ, (b, e) P3HT/AuNP NF: PCBM and (c, f) P3HT/AgNP NF: PCBM active layers. NP addition was made to 17.5 wt.% AgNO_3_ or AuNPs (including capping) wrt P3HT. g,h) Transient signal decays of g) the (0–1) GSB and h) excitons/polarons at 950 nm probe wavelength, normalized to the greatest ΔOD intensity after photoexcitation, presenting slower relaxation in the presence of AgNPs. i) Steady‐state PL of NP‐containing active layers, relative to the pristine nanofibrous active layer. NFs were electrospun for 120 s in each case.

For plasmonic, nanofibrous active layers to boost eventual OPV performance beyond the BHJ or pristine fibrous architectures, it is imperative that NP addition does not introduce recombination mechanisms. The resulting exciton or charge quenching can prevent the translation of LSPR‐enhanced exciton populations into correspondingly high polaron/carrier populations in the BHJ. In **Figure** **5**a–c, false color heat maps of the BHJ and Ag/AuNP‐containing P3HT NF:PCBM active layers are presented, with extracted line‐cuts in Figure 5d–f.

Normalized (0–1) GSB (≈560 nm probe, proportional to initial photoexcitation and the exciton/polaron populations) and exciton/polaron (950 nm probe) signal relaxation kinetics are displayed in Figure 5g,h, respectively (PP signal relaxation at 660 nm, Figure S24a, Supporting Information). Upon initial inspection, the pristine and AuNP‐containing fibrous active layers possess similar decay to the BHJ. Meanwhile, AgNP introduction slows the signal decay rate significantly. Likewise, a lesser AgNP addition produced a marginal reduction, see Figure S24b–d (Supporting Information). Slower decays indicate either increased photoinduced species’ lifetimes and/or a greater polaron generation rate and are in contrast to previous reports. Bare and oleylamine‐capped AgNPs have previously induced faster transient exction/polaron signal relaxation, owing to charge trapping, accumulation, and ultimately, quenching.^[^ ^28^, ^29^, ^85^ ^]^ Therefore, nanofibrous active layers are promising for harnessing the full potential of plasmonic NP addition.

Fitted time constants/amplitudes are similar in pristine thin‐film and fiber‐based layers, however, Table S2 (Supporting Information) further conveys the NP influence upon the photoexcited species. The fibrous heterojunction possesses enhanced polaron populations in the presence of AgNPs, as A_3_ rises from 28.9% (BHJ) to 39.6% absolute (a 37% relative improvement). Whilst longer polaron lifetimes are fitted with both NP types, they are extrapolated beyond the temporal window of 3 ns. Table 1 estimates that AgNPs generate a 46% larger free carrier population persisting for ≥1 ns, per exciton, relative to the BHJ.

Compounded with the harvesting enhancements reported within our previous study,^[^ ^16^ ^]^ the P3HT/AgNP NF:PCBM active layer contains 116% more polarons at 1 ns. Despite a 9% reduction in the polaron population per exciton in the AuNP‐containing active layer versus the BHJ, once additional photon absorption is considered, the active layer still contains 43% additional polarons at this time delay. The initial exciton generation enhancement is successfully translated into an enhanced density of free carriers.

Assuming accurate fitting, exciton lifetimes were also longer, perhaps due to a reduction in exciton recombination, dissociation, or exciton‐exciton annihilation. In Figure 5i, steady‐state NF PL before PCBM infiltration reveals that despite equal electrospinning time, the NP‐containing fibers produce 47% (AgNP) and 34% (AuNP) lower integrated PL. Thus, the metal cores may be involved in exciton quenching, either by increasing detrimental recombination or useful dissociation into free charges. With a flux of 70 µJ cm^−2^, some non‐negligible rate of bimolecular recombination is expected. However, as NP‐containing architectures harvest additional photons, one would expect the annihilation rate to be greater. This leaves the possibility of a reduced rate of exciton recombination for future study.

We suggest that NP addition to the nanofibrous active layer is not parasitic for two reasons. First, insulating ligands such as oleylamine or PEO, could provide electrical isolation from the active layer to prevent charge trapping. Due to the AuNP synthesis in neat oleylamine, the capping layer may be denser or thicker than in previous reports where trapping was observed.^[^ ^28^ ^]^ This observation would be consistent with previous works wherein the appropriate organic ligands (or ultrathin oxide layers) have been demonstrated to act as insulating barriers,^[^ ^85^, ^86^, ^87^ ^]^ and increasing the thickness of the capping layer reduces the probability of exciton or charge quenching.^[^ ^30^, ^88^ ^]^ Additionally, within a BHJ, the NPs are in contact with both the donor and acceptor and reports have suggested that the NP‐assisted recombination events occur at the interface or involve fullerene molecules.^[^ ^28^, ^29^ ^]^ Although we report evidence of PCBM diffusion, sequential deposition of the components may allow for a reduction in NP‐fullerene interactions via spatial separation, ameliorating these mechanisms. If plasmonic, nanofibrous devices can be optimized for efficient charge extraction, these novel active layers offer an exciting alternative to the BHJ.

## 3 Conclusions

A novel OPV architecture, the plasmonic, nanofibrous active layer, is produced by electrospinning ultra‐thin and continuous P3HT NFs containing nanoparticles, subsequently infiltrated with PCBM. We provide perhaps the most comprehensive evaluation of the potential performance of fibrous active layers to date by investigating charge carrier separation and lifetimes and predicting the charge transport capabilities via morphological and nanostructural studies. In the first application of low‐dose 4D‐STEM to electrospun fibers, we observe the shape, orientation, and distribution of crystallites in single electrospun P3HT NFs. Coupled with orientation‐dependent 2D GI‐XRD, we find that P3HT crystallites, assembled in solution before electrospinning, are aligned during spinning with the [0k0] axis oriented along the fiber. We envisage opportunities for enhanced in‐ and out‐of‐plane charge transport with π–π persistence lengths up to 80 nm, and a population of end‐on crystallites. 95%+ of PL emission is quenched following PCBM infiltration to form the fibrous active layer. This verifies that the size of the donor domains, templated by the fibers, are appropriate for charge separation, despite their diameters being significantly larger than the materials’ exciton diffusion length. Employing fs‐TAS, we confirm PL quenching results in free charge carriers, with a 15‐fold increase in polaron populations (per exciton formed, measured at 1 ns) with PCBM backfill. AgNP addition substantially slows the exciton/polaron signal decay. 1 ns post‐excitation, this active layer contains 45% more polarons than the BHJ, while polaron lifetimes could be extended. Typically, plasmonic BHJs exhibit faster TAS signal decay as metal NPs induce recombination. Our findings suggest that this active layer has significant potential for realizing greater OPV efficiencies versus thin films. As a sequentially deposited system, it offers control over the domain size and distribution and enables selective placement of additives into individual domains. This contribution indicates a high efficiency potential for this novel heterojunction, which can be simply translated to contemporary donor: acceptor systems.

## 4 Experimental Section

### Materials

Poly(3‐hexylthiophene‐2,5‐diyl) (P3HT, Mw 50 000) was obtained from Rieke Metals, and [6,6]‐phenyl‐C_61_‐butyric acid methyl ester (PCBM) from Ossila. Poly(ethylene oxide) (PEO, Mw 900 000), chloroform, *N,N*′‐dimethylformamide (DMF), silver nitrate (AgNO_3_), tetrachloroauric(III) acid trihydrate (HAuCl_4_.3H_2_O), oleylamine (technical grade, 70%), isopropanol (IPA) and methanol were all obtained from Sigma Aldrich. Solvents were dehydrated/degassed, and materials were stored under an inert atmosphere/in the dark.

### Nanoparticle Synthesis

AuNPs 10(nm) were synthesized via thermal reduction of HAuCl_4_ in neat oleylamine, which acts as the reducing agent, solvent, and stabilizing ligand. Briefly, HAuCl_4_ (100 mg) dissolved in oleylamine (2 mL) was injected into oleylamine heated to 175 °C (15 mL, 32 mmol) under nitrogen. A red‐black dispersion was formed and after 10 minutes, the reaction was cooled to ambient temperature. AuNPs were precipitated with methanol (50 mL) and pelletized by centrifugation. After removal of the supernatant and washing with additional methanol, the AuNPs were redispersed in chloroform to 10 mg mL^−1^. 3 nm AgNPs were produced within the spinning solution following AgNO_3_ addition as outlined in the following section. AgNO_3_ undergoes reduction by PEO (and DMF) over 96 h of spinning solution aging at 40 °C.

### Electrospinning of Pristine and NP‐containing P3HT NFs

P3HT (0.20 wt.%) and PEO (0.35 wt.%) spinning solutions were produced by first dissolving P3HT and PEO in chloroform at 40 °C for 1 h. For pristine P3HT NFs, DMF was added dropwise followed to produce a 95:5 w/w chloroform: DMF solution. For NP‐containing NFs, AgNO_3_ (yielding AgNPs) was dissolved in the DMF co‐solvent prior to addition, or AuNPs dispersed in chloroform were directly added. Final NP concentrations were made to 0.035 wt.% AgNO_3_ or AuNPs (including the capping layer). In the case of AuNPs, DMF could be omitted. The solution was then aged at 40 °C whilst stirring for up to 96 h.

The spinning solution was loaded into a syringe and fed at 1 mL h^−1^ through a 27G flat‐tip stainless steel needle using a syringe pump. A 23 kV voltage bias was applied between the needle and a grounded steel plate, separated by a distance of 20 cm. Electrospinning was conducted under an inert atmosphere at 20–22 °C, however, fibers could be spun in the air at a relative humidity of <30 RH%. The purple NFs were then washed in IPA at 70 °C for 1 h to selectively dissolve PEO, and dried under nitrogen flow.

### Preparation of the Plasmonic, Nanofibrous Active Layer

ITO glass substrates (20 Ω sq^−1^, Ossila) were prepared by ultrasonic cleaning in a dilute, aqueous detergent solution for 15 minutes, repeated with ethanol, IPA, and acetone. Finally, the surface was treated with UV Ozone for 15 minutes before placing it on top of the grounded collector. NFs were electrospun for 120 s and processed as described above. Nanoweb infiltration was achieved by spin‐coating a saturated PCBM solution in DCM (14 mg mL^−1^). The solution (30 µL) was dispensed onto the nanoweb at low speed (250 rpm, 5s) before ramping to 2500 rpm (2500 rpm s^−1^). The first step was used to provide additional time for the solution to infiltrate the nanoweb. Reference BHJs were produced by spin‐coating a 1:1 P3HT:PCBM solution (40 mg mL^−1^ in CB) at 1 500 rpm for 40 s.

### Characterization—Microscopy

Nanofibers and active layers, sputter coated with 6 nm Pt, were imaged using a Zeiss Merlin FEG scanning electron microscope (SEM) under an operating voltage of 6 kV. STEM‐in‐SEM imaging was conducted using a Zeiss a‐STEM detector at an accelerating voltage of 20 kV. Samples for STEM analysis were prepared directly on lacey carbon transmission electron microscopy (TEM) grids. NF diameters (width and heights) were determined from SEM and AFM micrographs, respectively, using 100 unbiased counts.

### 4D‐STEM

4D‐STEM was acquired using a JOEL ARM 300 CF fitted with ultra‐high‐resolution pole piece, cold FEG, and double aberration correction (Diamond Light Source, UK). The instrument was operated at a 300 kV accelerating voltage at a low electron fluence. The nanobeam configuration was formed by turning off the aberration corrector at the probe‐forming optics and using a 10 µm aperture to obtain a probe convergence semi‐angle of ≈0.8 mrad (determined by a py4DSTEM^[^ ^43^ ^]^ thresholding algorithm). This provides ≈3 µm depth of focus, sufficient to fully sample the NF (equation given by Nellist^[^ ^89^ ^]^). An electron dose of < 6 e^−^ Å^−2^ was calculated with a probe current of ≈0.4 pA, measured using a Faraday cup, and a dwell time of 1 ms pixel^−1^. The nanobeam was raster scanned over the sample at 256 × 256 positions and a 2D diffraction image (512 × 512 pixels) was recorded using a quad‐chip Merlin Medipix pixelated direct electron detector (Quantum Detectors) at a camera length of 40 cm for each probe position. Data was processed by using the workflow discussed within the main text using py4DSTEM (0.14.8). Python code was adapted from publicly available notebooks provided on the py4DSTEM GitHub repository. Fibers were directly electrospun onto a lacey carbon TEM grid (Agar Scientific) and PEO was removed by submerging the grid in IPA at 70 °C. Using an Au cross‐grating reference (Ted Pella), the diffraction and real‐space pixel sizes were calibrated, alongside an affine transformation for elliptical distortion correction to ensure diffraction roundness. The relative reciprocal‐to‐real space rotation was determined using a highly defocused shadow image of the cross‐grating. Features were tracked while scanning to determine the probe raster direction. Calibrations were then applied to the fiber datasets and Bragg peaks were detected using a cross‐correlation template matching protocol with a synthetic probe template. Finally, crystalline phase orientation mapping was conducted by identifying the Bragg peak of the highest cross‐correlation intensity in a selected region of the diffraction pattern at each probe position and assigning the probe position the angle between the *y*‐axis, and a line from the central disc to the Bragg peak.

### 2D GI‐XRD

2D GI‐XRD was measured with a Rigaku SmartLab X‐ray diffractometer equipped with a PhotonMax 9 kW rotating anode source producing Cu Kα radiation at a GI of 0.2° θ, and a HyPix‐3000 2D detector, to collect OOP and IP scattering simultaneously. OOP diffraction was collected between 0 and 30° 2θ_OOP_ at 5° 2θ_OOP_ min^−1^. The 2D detector was held at a sample‐to‐detector distance of 150 mm and was translated by +12° 2θ_IP_ to capture IP diffraction between −2–+26 ° 2θ_IP_. Incident end optics consisted of a parallel beam slit, 5 mm spot size height‐limiting slit, 1° IP parallel slit collimator, and knife edge. The aligned nanowebs were electrospun onto Si wafers using a rotating collector at ≈1000 rpm and scattering behavior was recorded when the fiber axes were oriented parallel and perpendicular to the incumbent X‐ray beam. IP and OOP profiles were extracted between 0–10° β and 77–87° β and azimuthal profiles were extracted over 0–87° β, averaging between 4–7° 2θ and 21–25° 2θ for (100) and (020) respectively. The influence of the optics and background subtraction methodology was described in the discussion of Figures S7 and S8 (Supporting Information). Determination of the degree of fiber and crystallite alignment by Herman's orientation factor applied to SEM micrographs and 2D GI‐XRD azimuthal traces, respectively, was described in Table S1 (Supporting Information).

### Steady‐State PL Quenching

Samples were photo‐excited using a 398 nm diode laser (PicoHarp, LDH‐D‐C‐405 M) on a continuous wave setting with a power density of 9900 mW cm^−2^. The PL resulting from excitation was collected and coupled to a grating spectrometer (Princeton Instruments, SP2558) which directed the spectrally dispersed PL onto a silicon iCCD (intensified charge‐coupled device, PI‐MAX4, Princeton Instruments) for detection. Samples were mounted in a vacuum cell at ∼10^−2^ mbar. PL quenching in fibrous active layers was determined by comparing the PL before and after PCBM infiltration. Quenching in the BHJ was estimated by comparing the radiative emission of a P3HT thin‐film with a P3HT:PCBM BHJ – produced by spin‐coating thin films from a 20 mg mL^−1^ P3HT solution in CB, and 20 mg mL^−1^ P3HT: 20 mg mL^−1^ PCBM solution in CB, respectively.

### Femtosecond‐TAS

fs‐TAS studies were conducted upon fiber and film‐based active layers fabricated upon z‐cut quartz substrates. Fibers cannot be easily electrospun onto insulating substrates, therefore a gap collector was used to produce a self‐supporting nanoweb through which the quartz was lifted. Pump and probe beam fractions were produced from 12 W pulses (1 kHz, 40 fs, 800 nm) generated by a Ti‐sapphire regenerative amplified laser system (Spectra‐Physics Dual Ascend Pumped Spitfire Ace) seeded by a Mai Tai (Spectra‐Physics).^[^ ^90^ ^]^ The pump fraction was tuned to 520 nm using an optical parametric amplifier (TOPAS‐prime with UV extension, Light Conversion). The pump pulse with a fluence of ≈70 µJ cm^−2^ was focused onto the sample, which was continuously blown with nitrogen. Passing 5% of a 1 W fraction through a CaF_2_ window generated a white light continuum (330 nm–1025 nm, a filter removed the 740 – 850 nm wavelength region to prevent detector overexposure by the 800 nm fundamental laser). A relative polarization between the pump and probe pulses was maintained at 54.7°. Pump‐probe delays between0.5 ps and 3 ns, at 40 fs resolution, were varied by translating a hollow gold retroreflector along a motorized stage (Newport M‐IMS500CCHA). Changes to optical density (∆OD) were determined using a fiber‐coupled spectrometer (Avantes, AvaSpec‐ULS1650F) from dark backgrounds and unpumped spectra. Spectra were chirp‐corrected using the KOALA^[^ ^91^ ^]^ package, and decays at key probe wavelengths were fitted using a tri‐component exponential decay function in OriginPro (Figure S24, Supporting Information discussion).

## Conflict of Interest

The authors declare no conflict of interest.

## Author Contributions

R.M.S., H.E.A., and N.G. developed this work and wrote the initial manuscript. R.M.S. conducted NP and NF synthesis, characterization, and analysis. K.A.E. performed PL quenching studies under the supervision of L.M.H. C.S.A., M.D., and G.T.T. helped design the 4D‐STEM experiment, and supported data analysis. J.M.W. and R.M.S. performed the fs‐TAS experiments. Supervision by B.M.M., H.E.A., and N.G. All authors contributed to the review and editing of the final manuscript.

## Supporting information



Supporting Information

## Data Availability

The data that support the findings of this study are available in the supplementary material of this article.
