# Supplementary material for: Nanostructure and Photovoltaic Potential of Plasmonic Nanofibrous Active Layers
Source: Small. 2024 Nov 22;21(3):2409269. doi: 10.1002/smll.202409269 (PMC11753493; doi:10.1002/smll.202409269)
Supplement: Supplementary file 1 — Supporting Information [file SMLL-21-2409269-s001.pdf]

NANO · MICRO
small

Supporting Information

for *Small*, DOI 10.1002/smll.202409269

Nanostructure and Photovoltaic Potential of Plasmonic Nanofibrous Active Layers

Ryan M. Schofield, Barbara M. Maciejewska, Karim A. Elmestekawy, Jack M. Woolley, George. T. Tebbutt, Mohsen Danaie, Christopher S. Allen, Laura M. Herz, Hazel E. Assender and Nicole Grobert**

Supporting Information

Nanostructure and Photovoltaic Potential of Plasmonic Nanofibrous Active Layers

R. M. Schofield[†,*], B. M. Maciejewska[†], K. A. Elmestekawy[‡], J. M. Woolley[§], G. T. Tebbutt, M. Danaie[||], C. S. Allen[†,||], L. M. Herz[‡], H. E. Assender[†] and N. Grobert[†,*]

[†]Department of Materials, University of Oxford, Parks Road, Oxford, OX1 3PH UK

[‡]Department of Physics, University of Oxford, Clarendon Laboratory, Parks Road, Oxford, OX1 3PU, UK

[§]Department of Physics, University of Warwick, Gibbet Hill Road, Coventry, CV4 7AL, UK

[||]electron Physical Science Imaging Centre, Diamond Light Source, Didcot, OX11 0DE, UK

Supporting Information

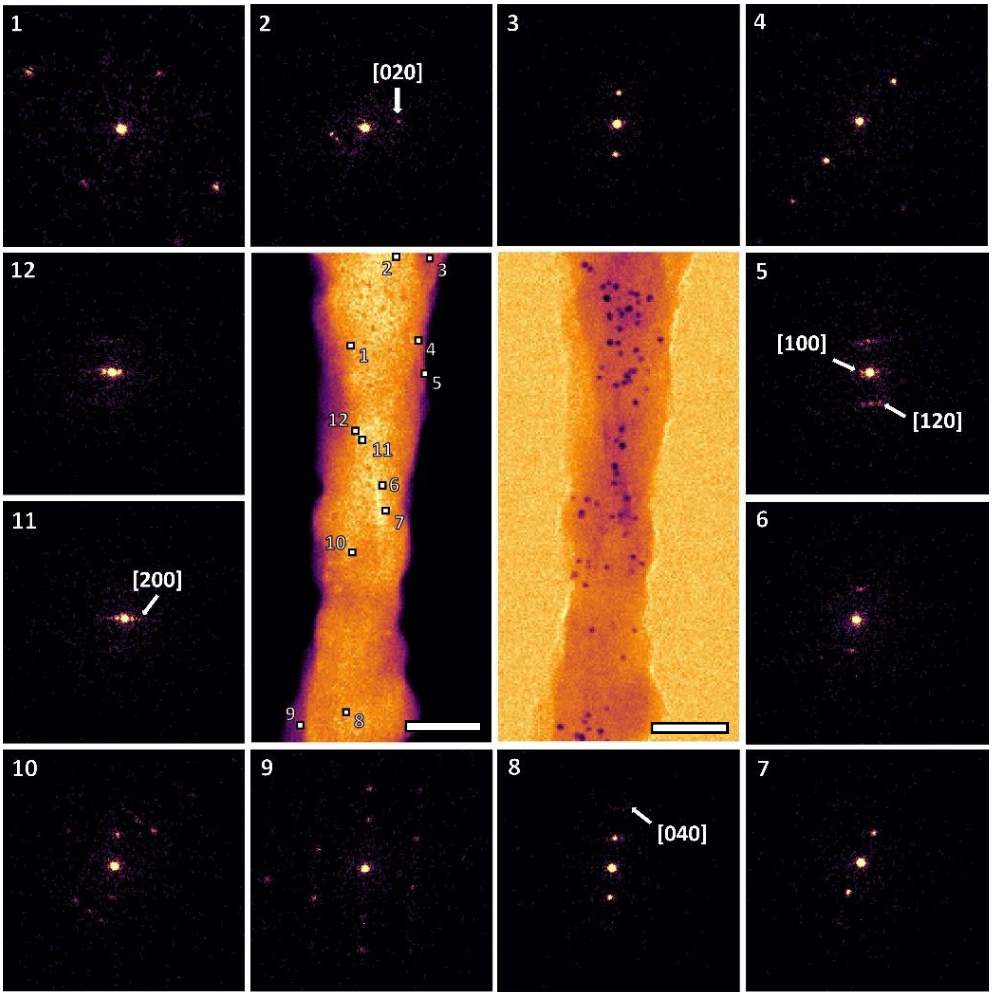

Figure S1 - Representative diffraction patterns produced at the probe position labelled in the P3HT/AuNP NF vDF (scale bar: 100 nm). The LHS vDF was integrated over the entire diffraction pattern, whilst the RHS vDF is produced by masking the central disc. Diffraction patterns have been corrected for real-to-diffraction space rotation (257° anti-clockwise).

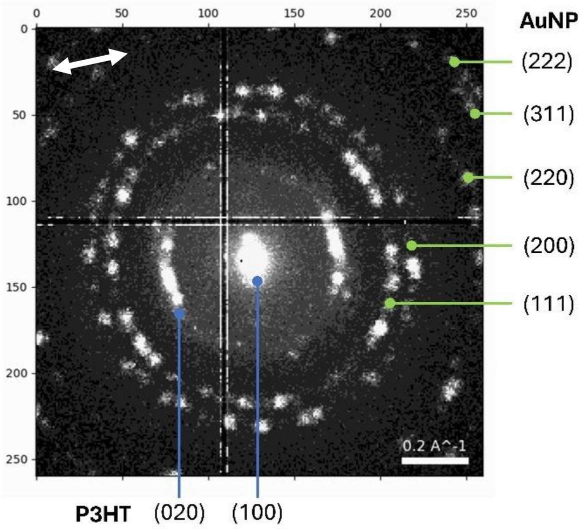

Figure S2 – Assigned P3HT and AuNP Bragg rings visible in the maximum diffraction pattern of the P3HT/AuNP NF. Arrow denotes the real-space fibre direction.

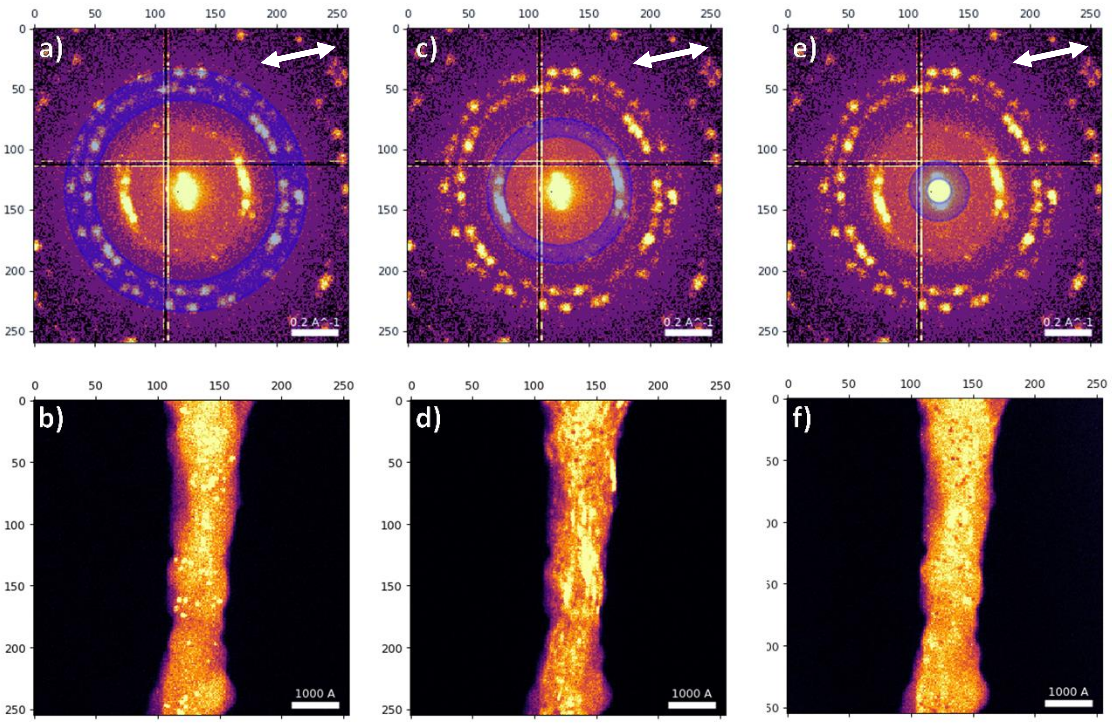

Figure S3 – (a, c, e) Maximum diffraction pattern presenting selected annular regions and **(b, d, f)** reconstructed vDFs of the P3HT/AuNP NF, revealing the distribution of the **(a, b)** (111) and (200) interplanar spacings of AuNPs, and **(c, d)** (020) π-π stacking and **(e, f)** (100) lamellar stacking of P3HT. A vDF is akin to a dark field image produced in SAED, formed from electrons diffracting through a scattering region of interest by the use of an aperture positioned in the diffraction plane. Arrow denotes the real-space fibre direction.

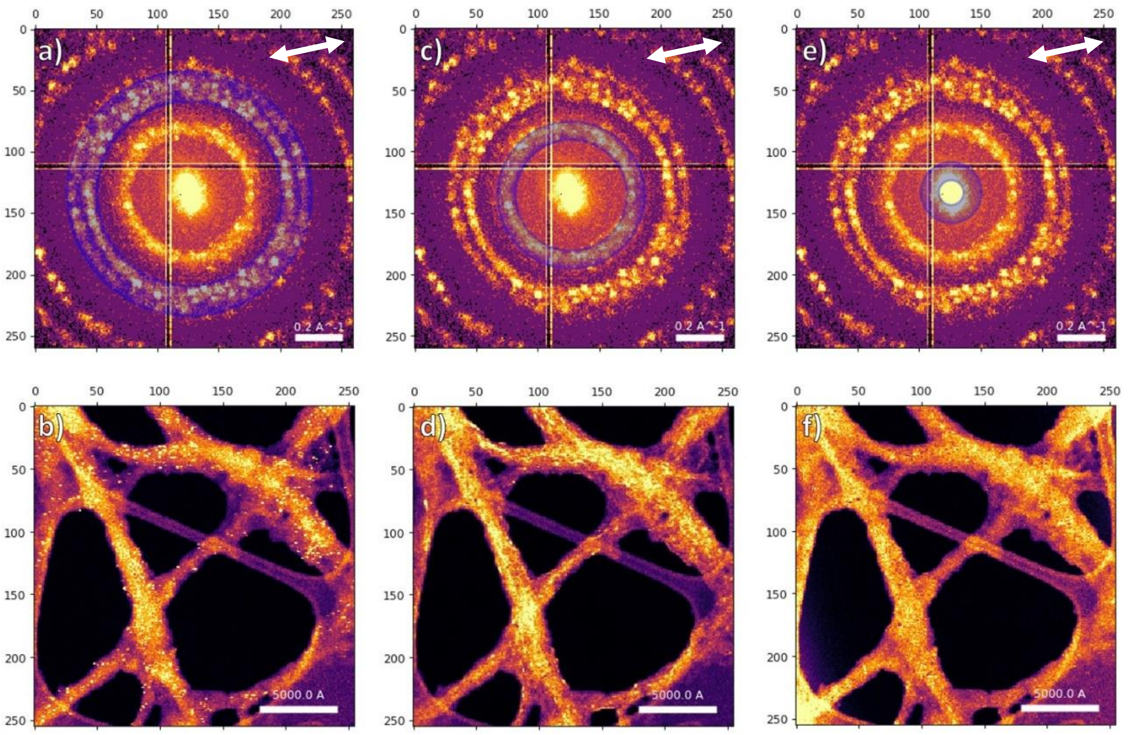

Figure S4 – (a, c, e) Maximum diffraction patterns presenting selected annular regions and **(b, d, f)** reconstructed vDFs of the P3HT/AuNP nanoweb featuring several NFs, revealing the location of **(a, b)** (111) and (200) interplanar spacings of AuNPs, and polymer **(c, d)** (020) π-π stacking and **(e, f)** (100) lamellar stacking. Arrow denotes the real-space fibre direction.

Supporting Information

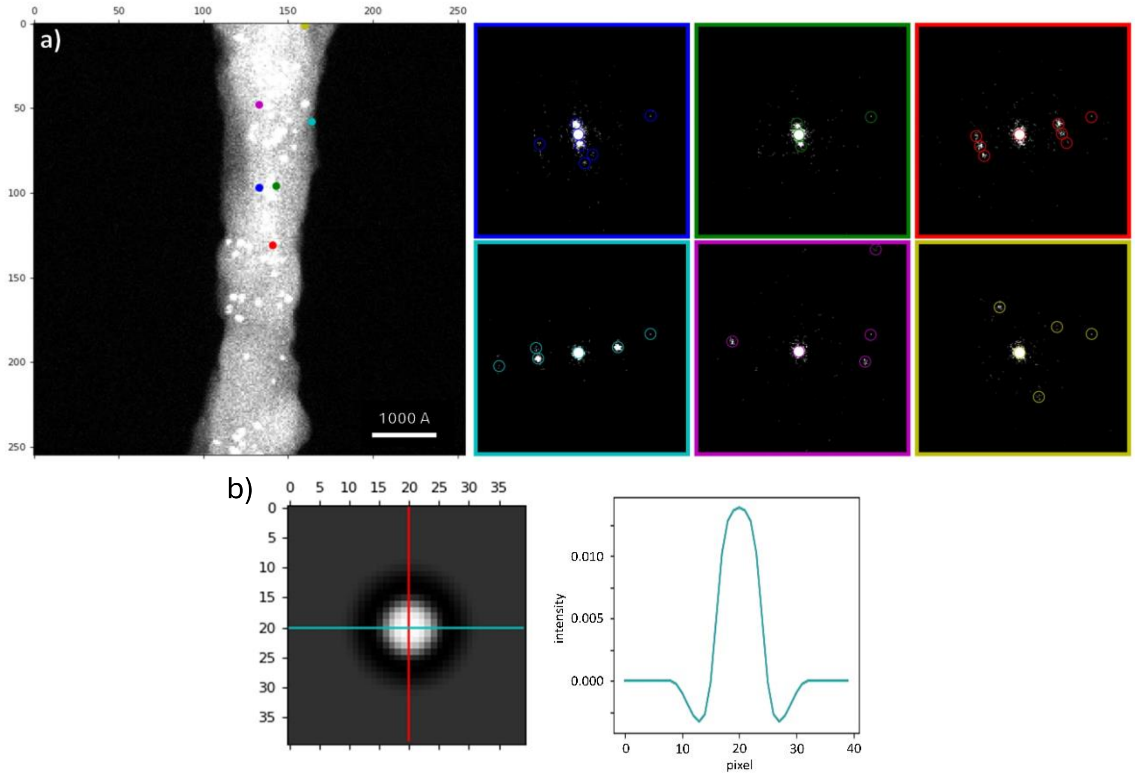

Figure S5 – (a) Diffraction patterns and **(b)** synthetic probe use for hyperparameter tuning of the cross-correlation Bragg detection protocol. The outline of the diffraction pattern refers to the colour coded probe positions annotated in the vDF.

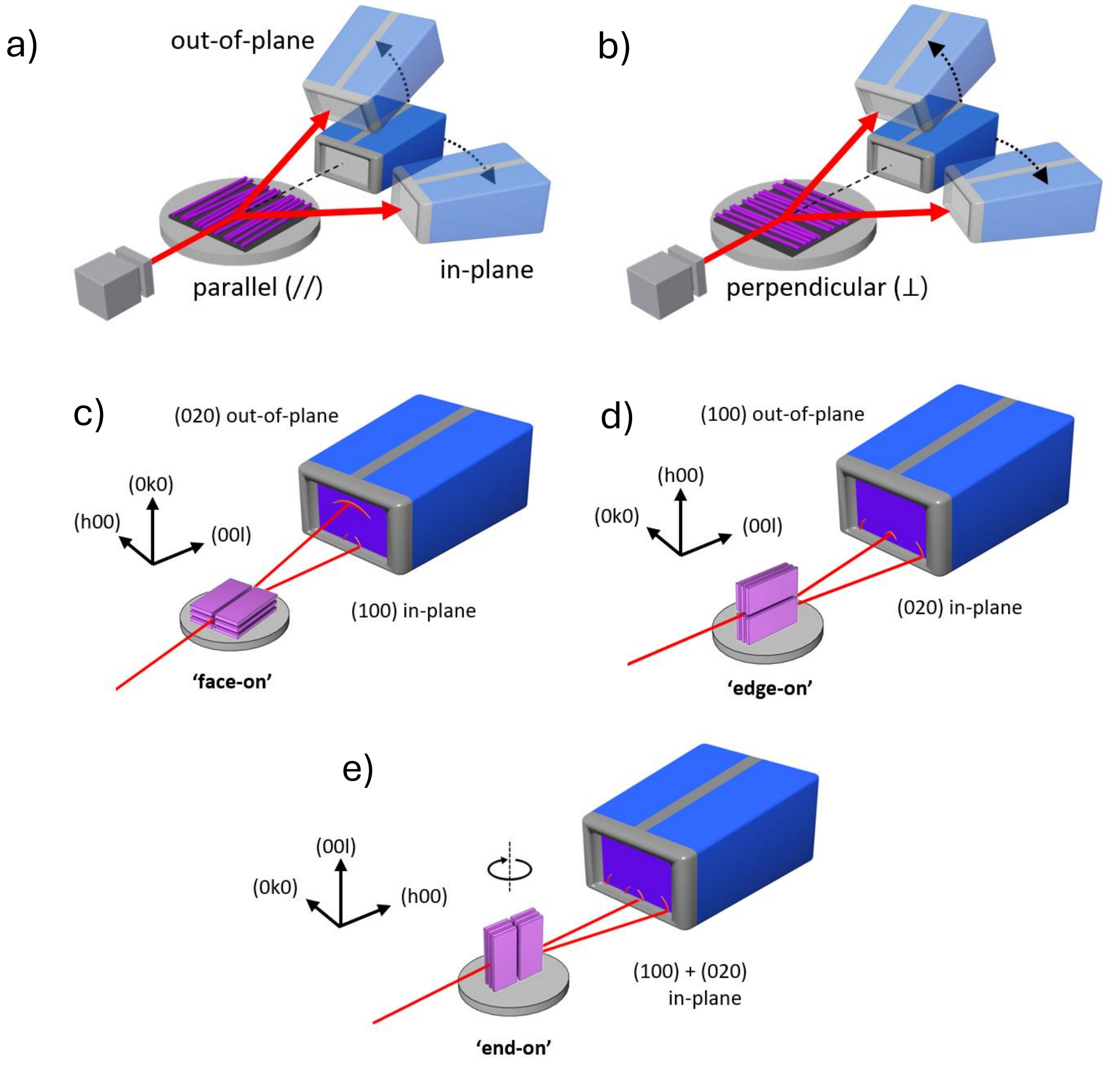

Figure S6 – (a) Parallel and **(b)** perpendicular fibre-to-beam orientations wrt an aligned fibre mat, collected using a rotating collector, used in 2D GI-XRD studies to elucidate the nature of crystallite alignment. Expected OOP and IP scattering patterns generated by the **(c)** face-on, **(d)** edge-on and **(e)** end-on crystallite configurations (wrt the collection substrate).

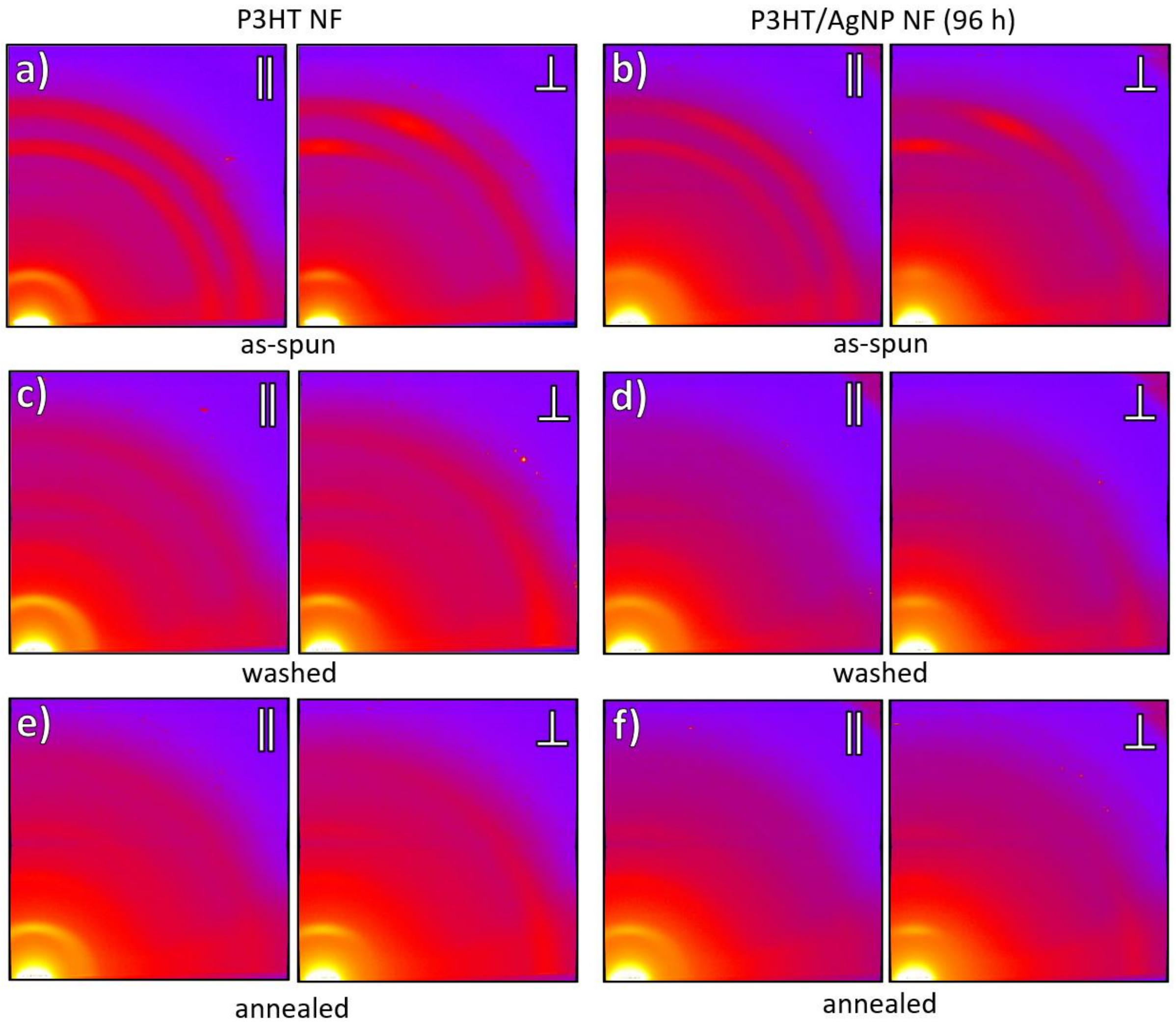

Figure S7 – 2D GI-XRD detector images of **(a, c, e)** pristine P3HT NFs and **(b, d, f)** P3HT/AgNP NFs, **(a, b)** as-spun (containing PEO) and **(c, d)** after PEO removal and **(e, f)** annealing at 120 °C for 20 minutes. Scattering characteristics were recorded under parallel (‖) and perpendicular (⊥) X-ray beam orientations wrt the fibre axis. Note that the radiation propagates along the substrate plane (0.2° GI), opposed to electrons, which were incident at a normal.

Supporting Information

Discussion

To capture the widest scattering view, sample-to-detector separation was minimised to 150 mm. The incident end optics comprised of a parallel beam slit, 5 mm spot size height-limiting slit and 1° IP parallel slit collimator. In **Figure S9**, it is clear that IP (100) scattering possesses a wider FWHM than at the OOP meridian. This arises due to collimation of the X-ray beam using a parabolic mirror, however this only produces a parallelised beam in the vertical direction, whilst IP, the beam is still divergent. The incident optics restrict divergence to 1°, however this means that peaks are inherently broader when scattering IP, particularly at low angles.

(020) azimuths are produced via background subtraction using the corresponding azimuth extracted from a bare Si wafer reference spectrum. For (100) scattering, the most effective approach was found to be subtraction of the 7- 7.5 ° 2θ azimuth (beyond the (100) peak) extracted from the same detector image, scaled using the 1D 2θ profile. Air-scattering intensity is greater IP than OOP, as in Figure S7a-b, due to the use of a knife edge to attenuate OOP scattering, shown in Figure S7c.

The detector was also used in 1D mode to measure the evolution of (100) scattering as the sample is rotated about 180° ϕ. Here, a 15 mm size limiting slit is used allowed full illumination of the sample throughout the ϕ rotation. The traces evolved for pristine and AgNP-containing P3HT NFs show invariance of the extent of (100) plane alignment to the reduction in fibre diameter.

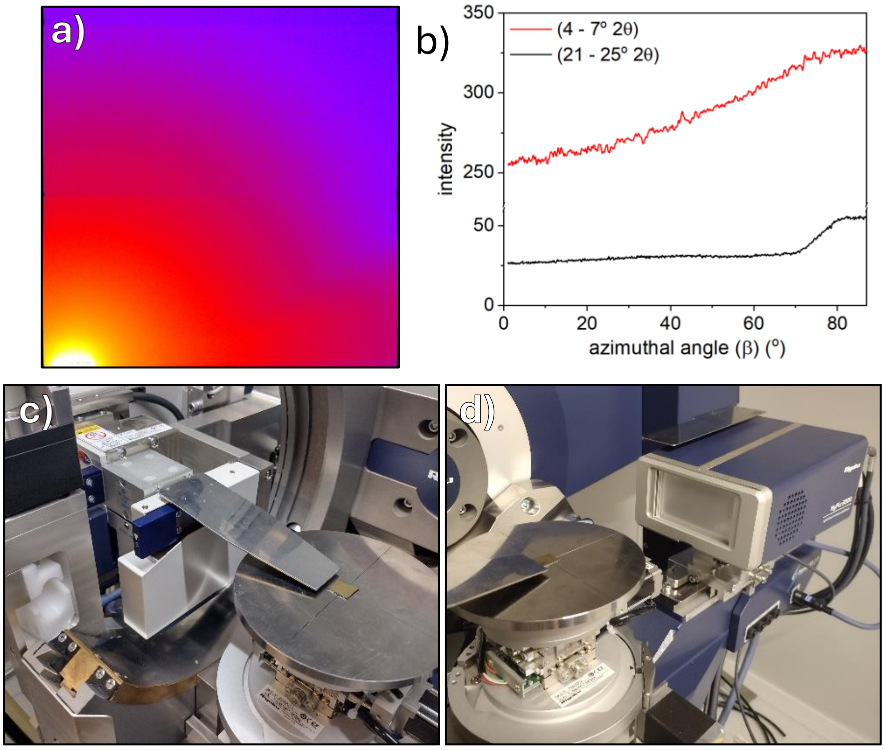

Figure S8 – (a) 2D detector image of a bare Si wafer and **(b)** azimuths (used for background subtraction) extracted at the (100) and (020) scattering angle showing greater air scattering along the IP axis. Digital photographs of 2D GI-XRD setup with **(c)** incident optics and **(d)** 2D detector.

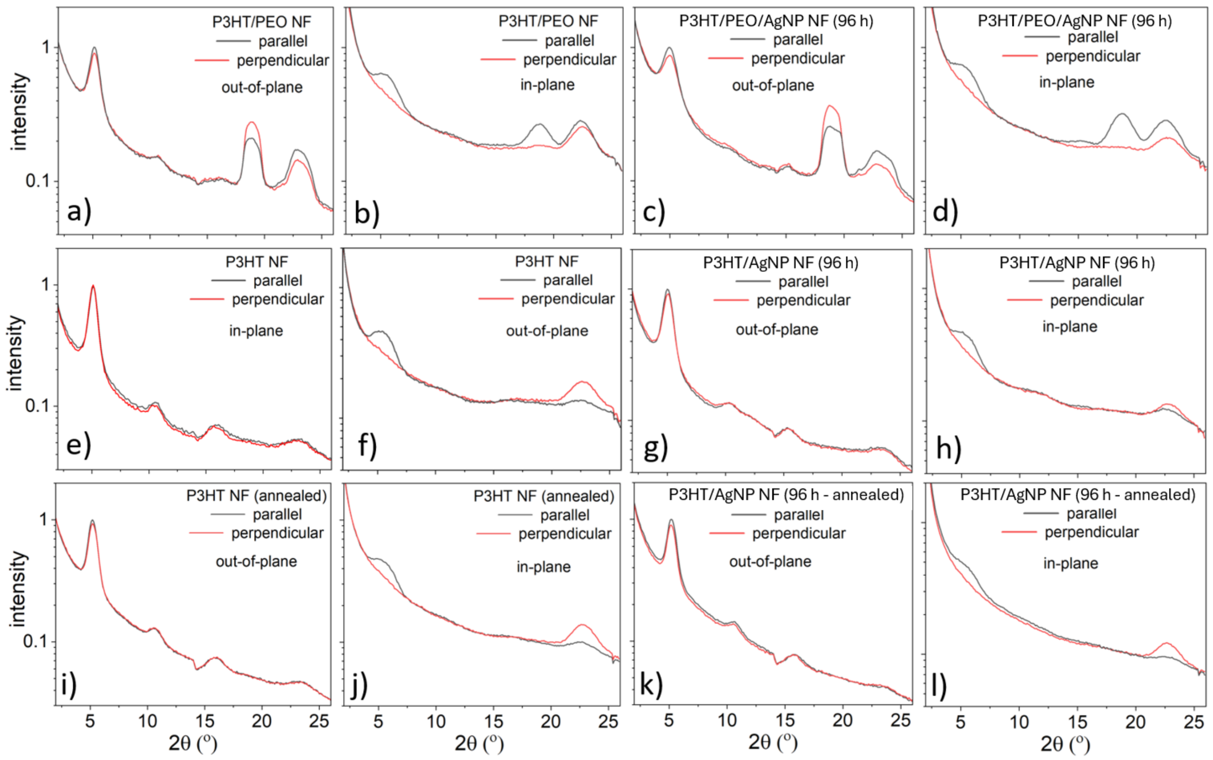

Figure S9 – 1D scattering profiles of **(a – d)** as-spun, **(e – h)** washed and **(i-l)** annealed **(a-b, e-f, i-j)** pristine and **(c-d, g-h, k-l)** AgNP-containing P3HT NFs, extracted from the detector images in Figure S7 along the **(a, c, e, g, i, k)** OOP (0 – 10 °β) and **(b, d, f, h, j, l)** IP axis (77 – 87 °β) under parallel (∥) and perpendicular (⊥) fibre axis to X-ray beam orientations.

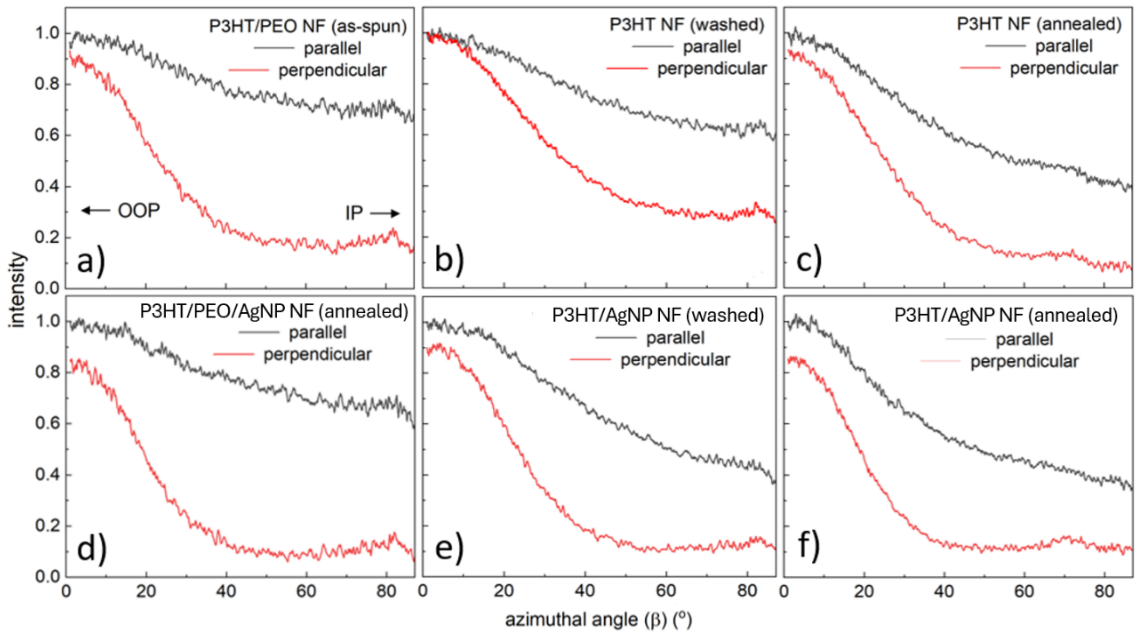

Figure S10 – (100) azimuthal profiles extracted from OOP (0 °β) to IP (87 °β) and between 4 – 7 ° 2θ from the detector images in Figure S7 under parallel (∥) and perpendicular (⊥) fibre axis to X-ray beam orientations. As the fibres as processed, by PEO removal and annealed, the parallel orientation reveals an enrichment of the edge-on configuration.

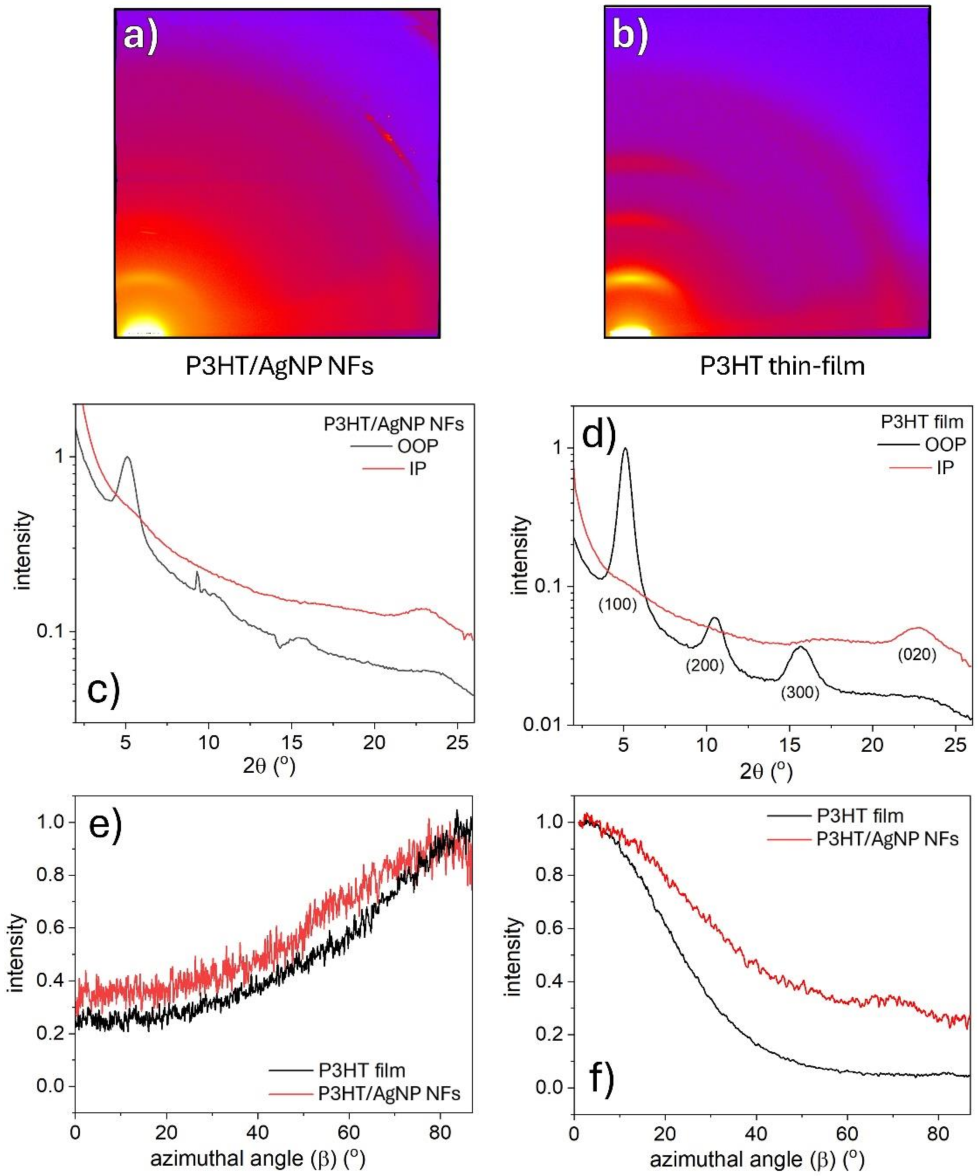

Figure S11 – (a) 2D GI-XRD detector image of a randomly-oriented web of P3HT/AgNP NF and **(b)** P3HT thin-film spin-coated from a chlorobenzene solution. 1D OOP and IP line-cuts extracted from the raw detector images of the **(c)** P3HT/AgNP NF and **(d)** P3HT thin-film. Azimuthal traces from OOP (0 °β) to IP (87 °β) of **(e)** (020) π-π and **(f)** (100) lamellar scattering generated by the nanoweb and thin-film.

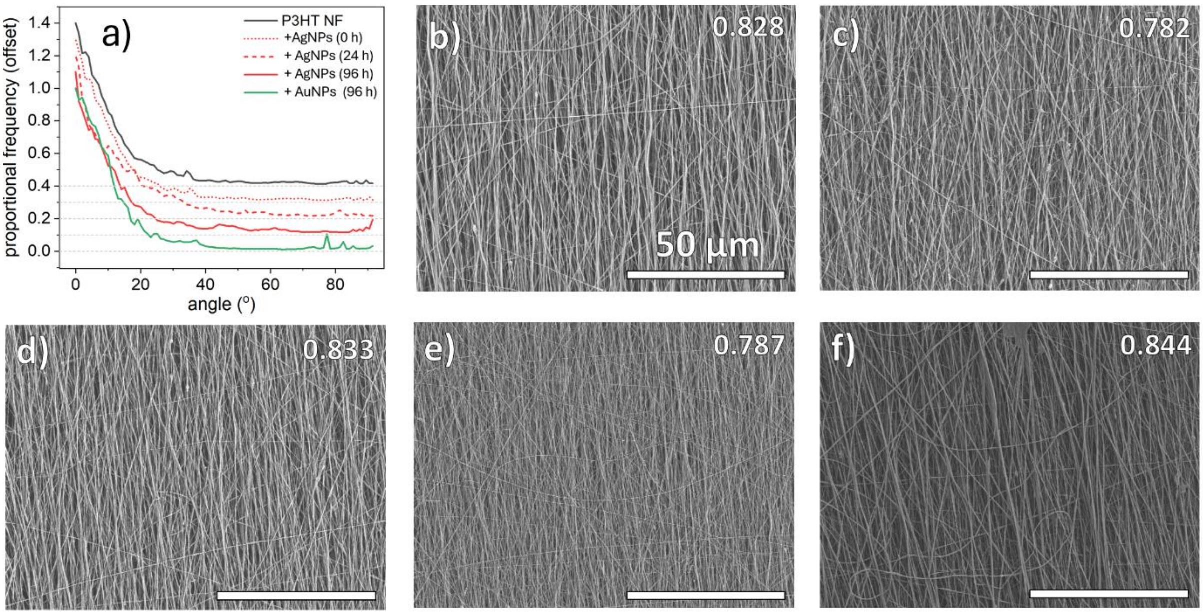

Figure S12 – (a) Histogram containing fibre angular frequencies used to determine fibre-to-fibre orientation parameters. SEM micrographs of **(b)** P3HT NFs spun after 96 h ageing, P3HT/AgNP NFs spun after **(c)** 0 h, **(d)** 24 h and **(e)** 96 h ageing, and **(f)** P3HT/AuNP NFs spun after 96 h ageing. The values presented in (b – f) represent the extent of fibre-to-fibre orientation.

NF	$<P_2>_o$ (100)		$<P_2>_o$ (020)		$<P_2>_f$	$<P_2>_c$ (100)		$<P_2>_c$ (020)	
Fibre-Beam	∥	⊥	∥	⊥	n/a	∥	⊥	∥	⊥
**P3HT (96 h)**	0.081	0.192	0.040	-0.265	0.828	0.098	0.232	0.048	-0.320
**P3HT/AgNP (0 h)**	0.075	0.144	-0.074	-0.071	0.782	0.096	0.184	-0.095	-0.091
**P3HT/AgNP (24 h)**	0.082	0.155	-0.051	-0.065	0.833	0.098	0.186	-0.061	-0.078
**P3HT/AgNP (96 h)**	0.141	0.327	0.021	-0.094	0.787	0.179	0.416	0.027	-0.119
**P3HT/AuNP (96 h)**	0.051	0.306	0.015	-0.059	0.844	0.060	0.363	0.018	-0.070

Table S1 – Overall and crystallite orientation factors determined from traces extracted along the (100) and (020) 2D GI-XRD scattering azimuths within P3HT-based NFs, and fibre-to-fibre orientation factors calculated from SEM micrographs.

Discussion

The extent of crystallite orientation was calculated for the (100) and (020) sets of planes using Hermans' orientation function, $< P_2 >$, as defined in Equation S1, applied to the corresponding 2D GI-XRD azimuthal traces.

$$< P_2 >_o = \frac{3 < cos^2(\phi) > -1}{2} \quad (\text{Eqn. S1})$$

Here, $< cos^2(\phi) >$ is given by:

Supporting Information

$$< cos^2(\phi) > = \frac{\int_0^{\frac{\pi}{2}} I(\phi) cos^2(\phi) \sin(\phi)\, d\phi}{\int_0^{\frac{\pi}{2}} I(\phi) \sin(\phi)\, d\phi} \quad \text{(Eqn. S2)}$$

The intensity, $I(\phi)$, can be derived from $I(\beta)$ information, the scattered intensity at the azimuthal angle °β, via a spherical trigonometric relation:

$$\cos(\phi) = \cos(\beta)\cos(\theta) \quad \text{(Eqn. S3)}$$

where θ is the Bragg angle and β is the azimuthal angle from the OOP meridian (0° β). In this analysis, perfect alignment of the (hkl) planes parallel to the substrate results in a factor of 1, while a perpendicular orientation produce a -0.5 value, and an isotropic sample yields 0.

The Hermans' orientation factor, $< P_2 >_o$, convolves the crystallite alignment and fibre-to-fibre alignment, $< P_2 >_f$ through the relationship:

$$< P_2 >_c = \frac{< P_2 >_o}{< P_2 >_f} \quad \text{(Eqn. S4)}$$

Fibre-to-fibre alignment factors were determined for each nanoweb by applying Equation S1 to an angular frequency histogram, generated from SEM micrographs of the aligned nanowebs using the ImageJ plugin, Directionality (see Figure S7). Since the $< P_2 >_f$ of each nanoweb was relatively consistent, the relative changes upon applying Equation S4 were largely inconsequential.

The figure of merit, depicted in Figure 2i, for crystallite alignment along the fibre axis, was calculated from the difference between the $< P_2 >_c$ determined with parallel and perpendicular fibre-to-beam orientations. This approach aims to account for the influence of edge-on configuration enrichment during thermal annealing by utilising both orientations simultaneously.

$$\Delta < P_2 >_c = < P_2 >_{c,\perp} - < P_2 >_{c,\parallel} \quad \text{(Eqn. S5)}$$

Supporting Information

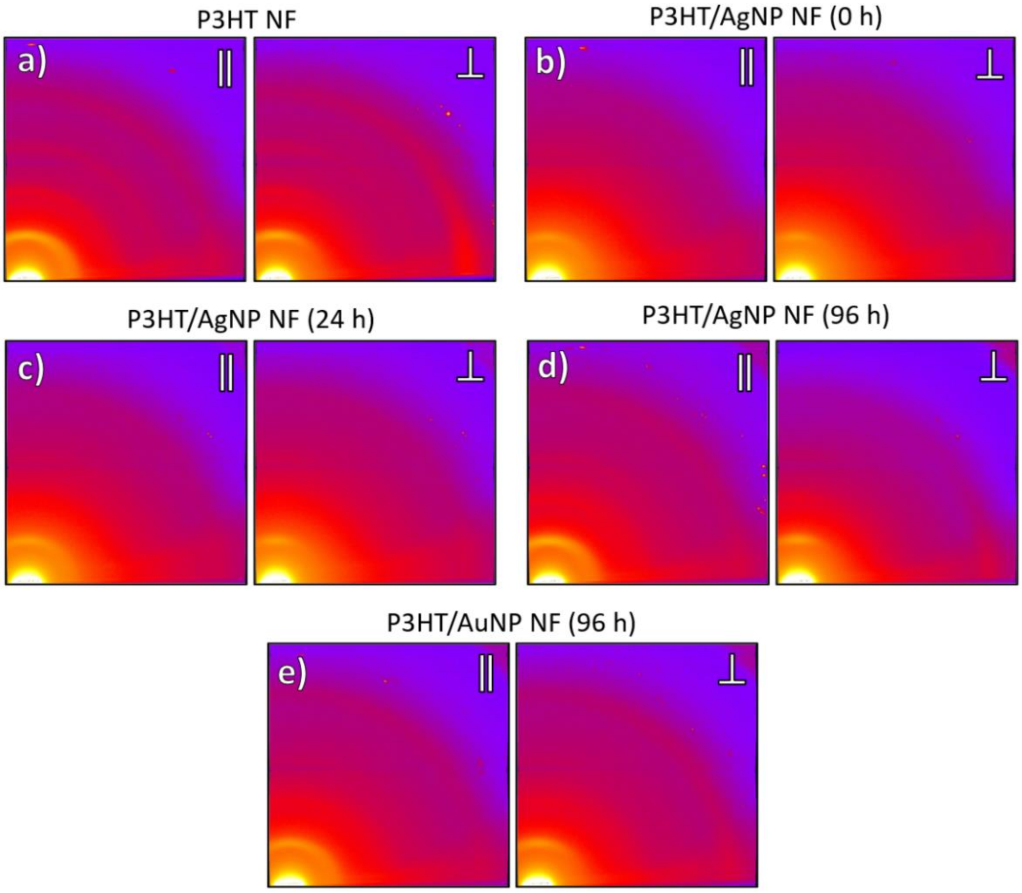

Figure S13 – (a) 2D GI-XRD detector images of **(a)** P3HT NF, **(b - d)** P3HT/AgNP NF and **(e)** P3HT/AuNP NF mats spun after **(b)** 0 h, **(c)** 24 h, and **(a, d ,e)** 96 h ageing.

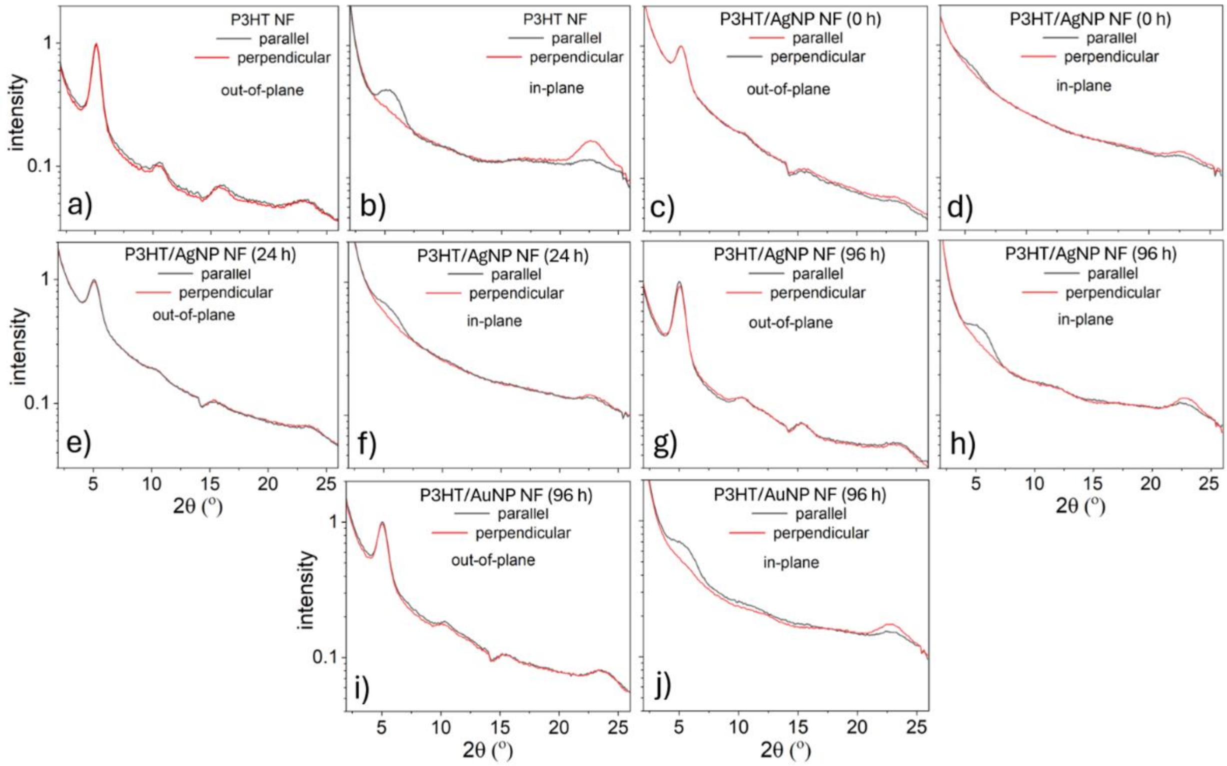

Figure S14 – 1D XRD profiles of **(a, b)** P3HT NFs, **(c - h)** P3HT/AgNP NFs spun after **(c, d)** 0 h, **(e, f)** 24 h and **(g, h)** 96 h of spinning solution ageing, and **(i, j)** P3HT/AuNP NFs spun after 96 h ageing, under parallel and perpendicular beam-fibre orientations, extracted along the **(a, c, e, g, i)** OOP and **(b, d, f, h, j)** IP axis.

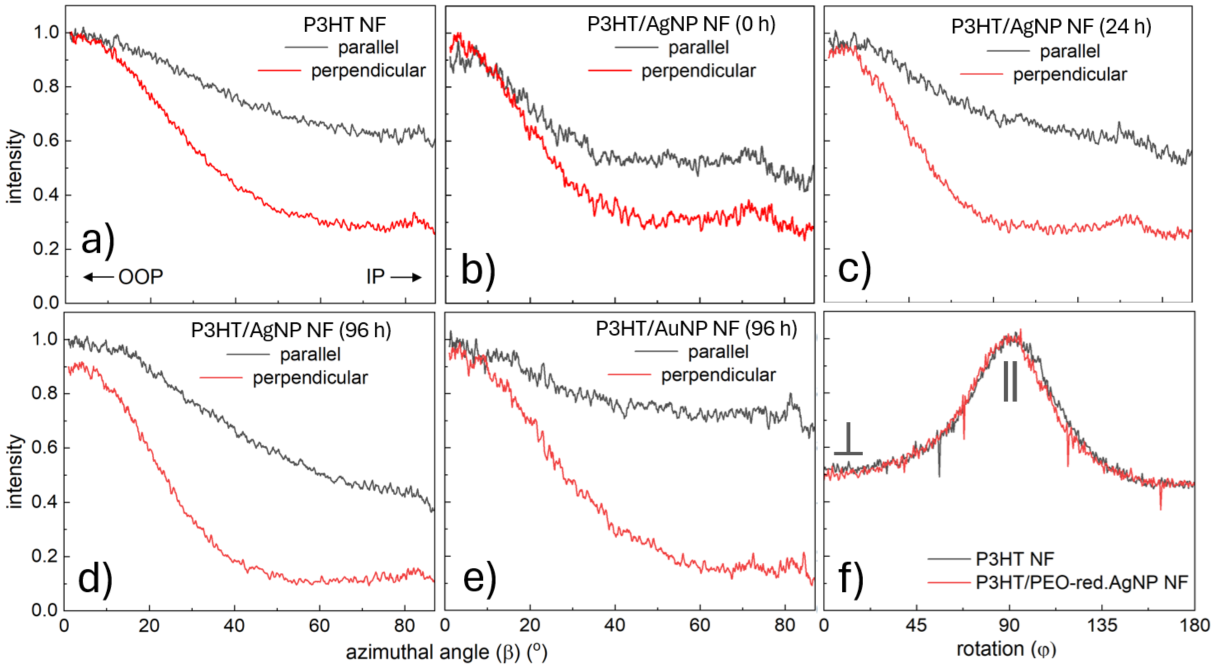

Figure S15 – **(a - e)** (100) azimuthal profiles of NFs from OOP (0 °β) to IP (87 °β), averaged between 4 – 7 ° 2θ, under parallel (∥) and perpendicular (⊥) fibre axis to X-ray beam orientations. **(f)** Evolution of (100) scattering intensity as thicker pristine and ultra-thin P3HT/AgNP NFs are rotated about a 180° ϕ rotation (⊥ at 0 and 180°, ∥ at 90°). The height limiting slit was increased to 15 mm to allow full illumination of the sample through the rotation.

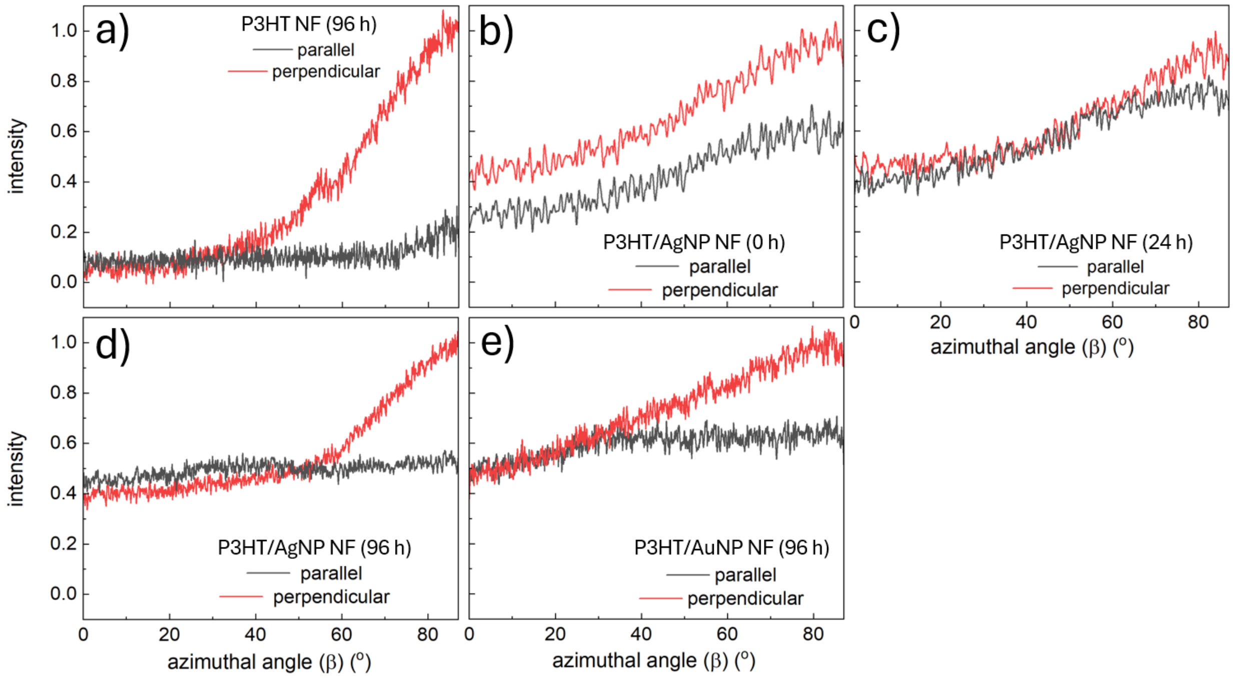

Figure S16 – (020) azimuthal profiles of NFs from OOP (0 °β) to IP (87 °β), averaged between 21 - 25 ° 2θ, under parallel (∥) and perpendicular (⊥) fibre axis to X-ray beam orientations.

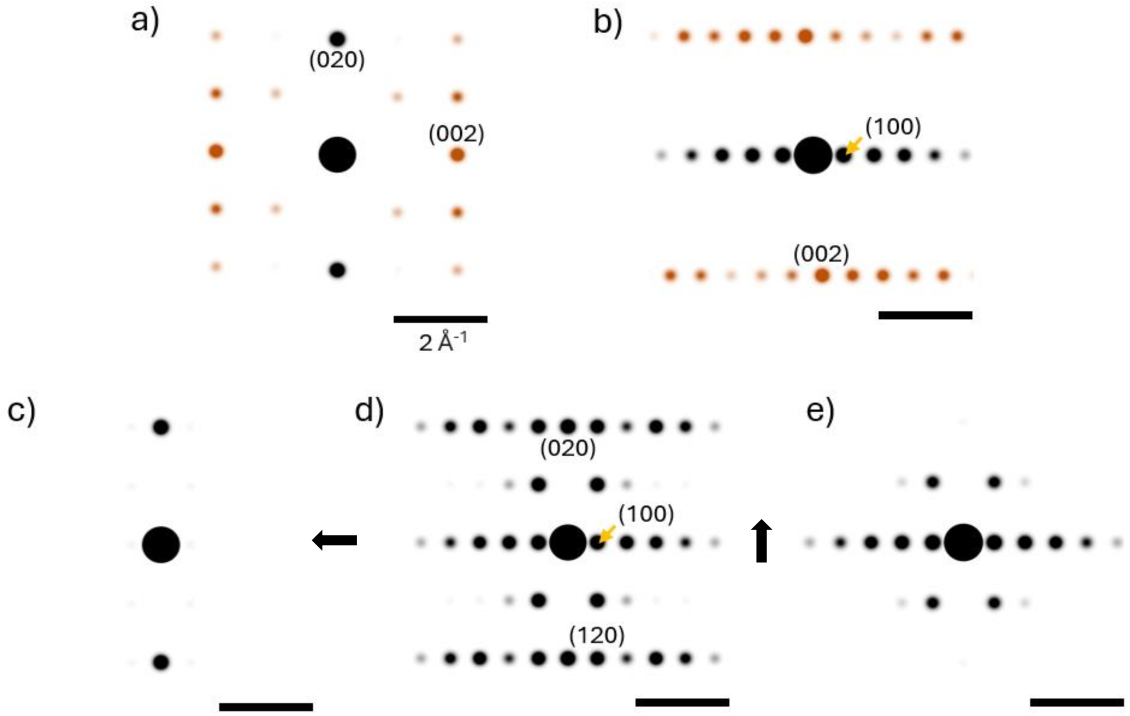

Figure S17 – (a) Simulated (SingleCrystal) electron diffraction generated when the beam is incident along the **(a)** (h00), **(b)** (0k0), and **(d)** (00l) axes. Orange spots are only produced with order in the (00l) direction. (100) and (020) attenuation is observed when the crystallite, initially viewed along the (00l) axis, is tilted beyond **(c)** 8° about the (0k0) axis and **(e)** 2° about the (h00) axis.

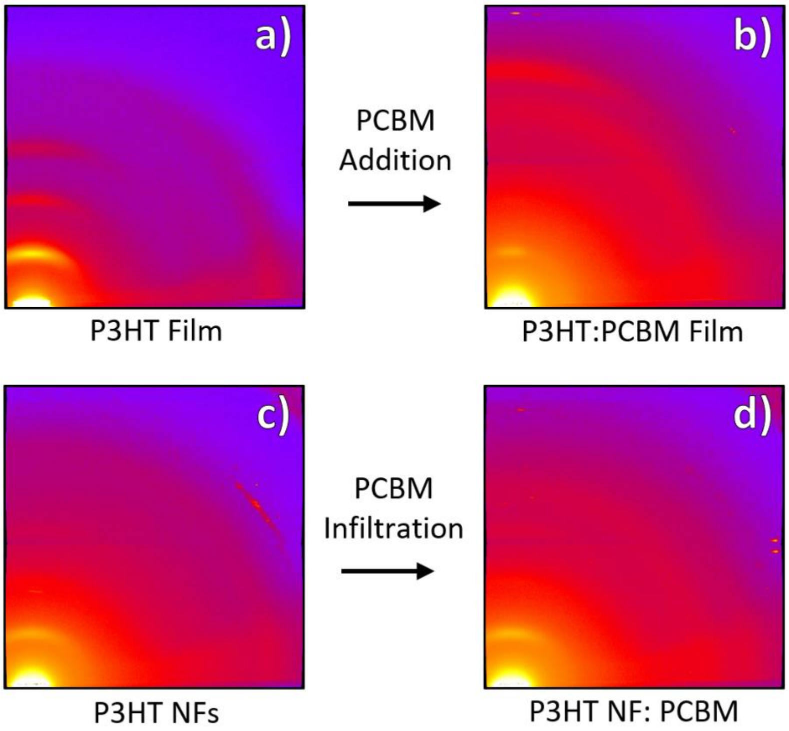

Figure S18 – 2D GI-XRD detector images of a **(a)** P3HT thin-film and **(b)** 1:1 P3HT: PCBM BHJ, presenting the disruption of edge-on preference upon blending with PCBM. Similar scattering behaviour is recorded for **(c)** P3HT NFs and **(d)** P3HT NF: PCBM after PCBM infiltration.

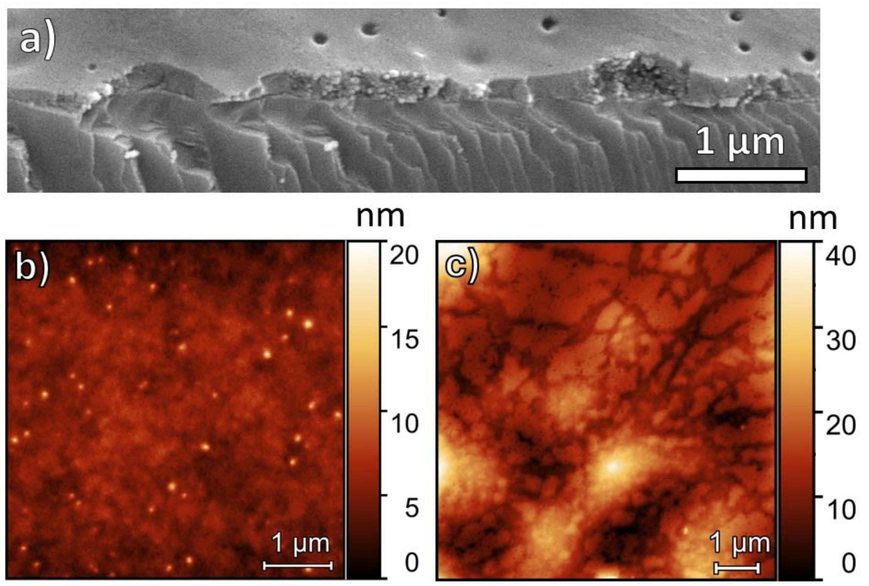

Figure S19 – **(a)** Cross-sectional SEM micrographs of P3HT NF: PCBM active layer revealing a thickness of 200 – 300 nm. AFM micrographs of **(b)** P3HT: PCBM BHJ and **(c)** P3HT/AgNP NF: PCBM active layers. Topology of the webs and active layers were analysed with an Agilent 5400 AFM in tapping mode.

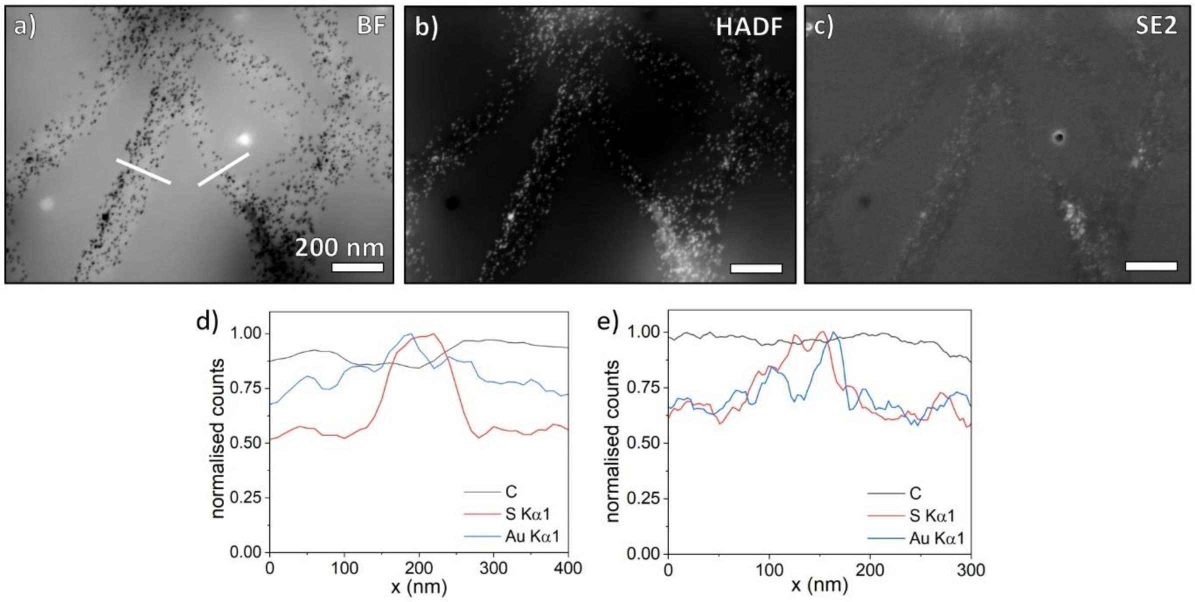

Figure 20 – (a – c) STEM-in-SEM and SEM micrographs of a low fibre density, P3HT/AuNP NF: PCBM active layer, collected in **(a)** Brightfield, **(b)** High-angle dark field and **(c)** SE2 modes. **(d – e)** EDX line scans show that P3HT is still localised to the NP pathways observed in (a – c) showing that fibre morphology is retained upon PCBM backfill.

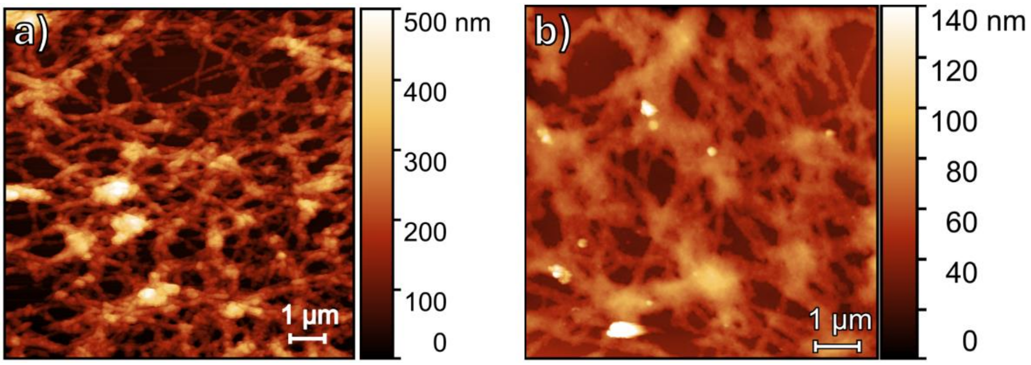

Figure S21 – AFM micrographs of the P3HT NFs **(a)** before and **(b)** after spin-coating with DCM (without PCBM) showing densification of the nanoweb during infiltration.

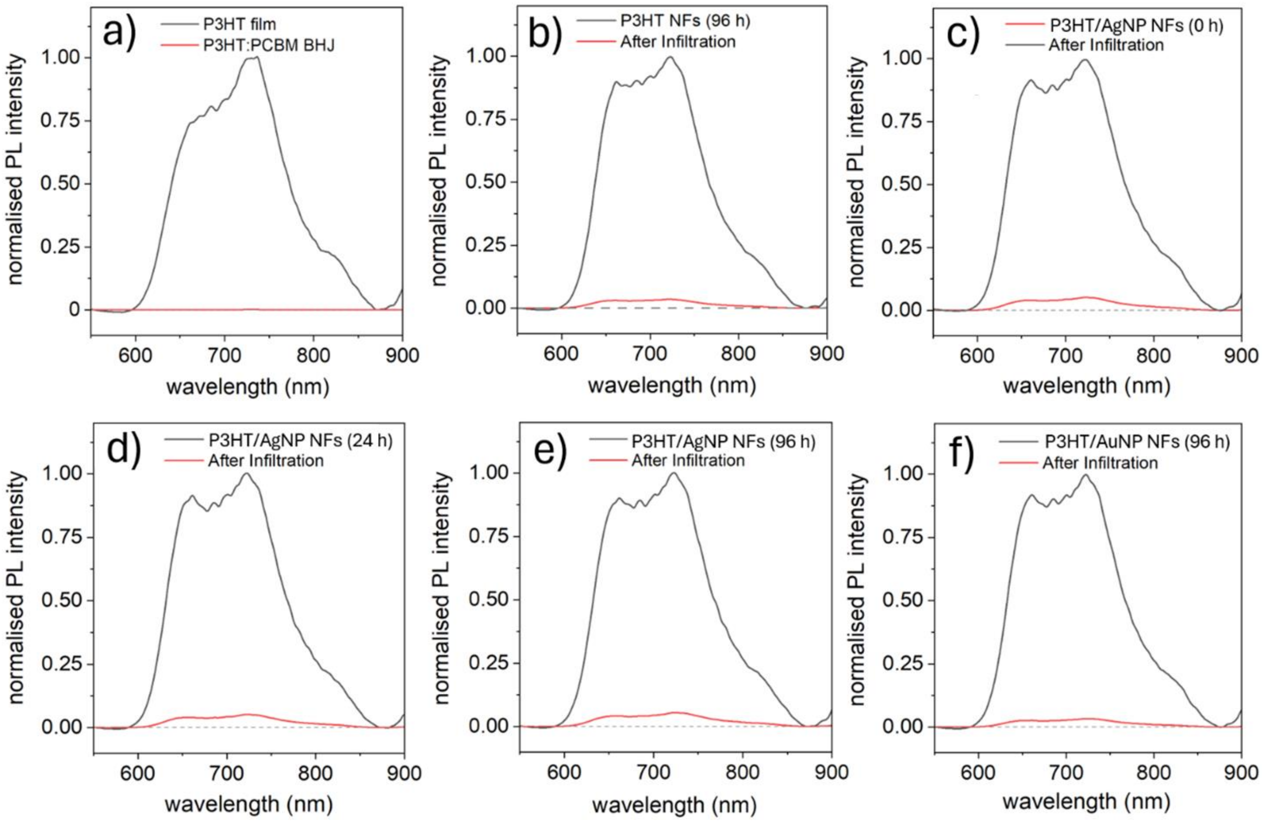

Figure S22 – PL quenching efficiency in a **(a)** 1:1 P3HT: PCBM BHJ versus P3HT thin-film, and quenching in **(b)** P3HT NFs, **(c - d)** P3HT/AgNPs spun after **(c)** 0 h, **(d)** 24 h and **(e)** 96 h ageing, and **(f)** P3HT/AuNP NFs spun after 96 h. Charge transfer efficiency to infiltrated PCBM was greater than expected in systems based upon thicker fibres (b – d), likely due to significant diffusion.

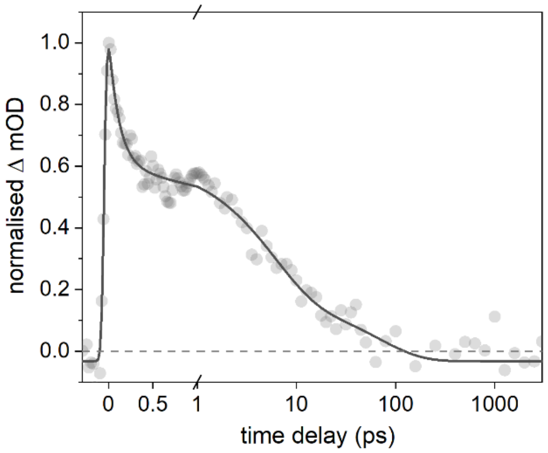

Figure S23 – (a) Relaxation kinetics of the transient signal of stimulated emission of P3HT excitons at a probe wavelength of 660 nm, pumped with a 520 nm excitation wavelength. Excitons in the P3HT NFs recombine within 100 ps.

Active Layer	A_1	τ_1 (ps)	A_2	τ_2 (ps)	A_3	τ_3 (ns)
**P3HT:PCBM BHJ**	0.22 (42.3%)	0.44 ± 0.04	0.15 (28.9%)	8.1 ± 0.8	0.15 (28.9%)	>3 (5 ± 0.6)
**P3HT NF (w/o PCBM)**	0.35 (62.5%)	0.59±0.05	0.14 (25.0%)	27.1±10.1	0.07 (12.5%)	0.43 ± 0.26
**P3HT NF:PCBM**	0.24 (47.1%)	0.46 ± 0.04	0.14 (27.5%)	7.5 ± 0.8	0.13 (25.5%)	>3 (4.4 ± 0.6)
**P3HT/AgNP NF:PCBM**	0.18 (34.0%)	0.65 ± 0.06	0.14 (26.4%)	21.9 ± 3.1	0.21 (39.6%)	>3(8 ± 1.6)
**P3HT/AuNP NF:PCBM**	0.28 (56.0%)	0.81 ± 0.04	0.11(22.0%)	22.9 ± 3.5	0.11 (22.0%)	>3 (18 ± 11)

Table S2 – Time constants and amplitudes of the tri-component exponential decay function fitted to the signal relaxation profiles at a probe wavelength of 950 nm.

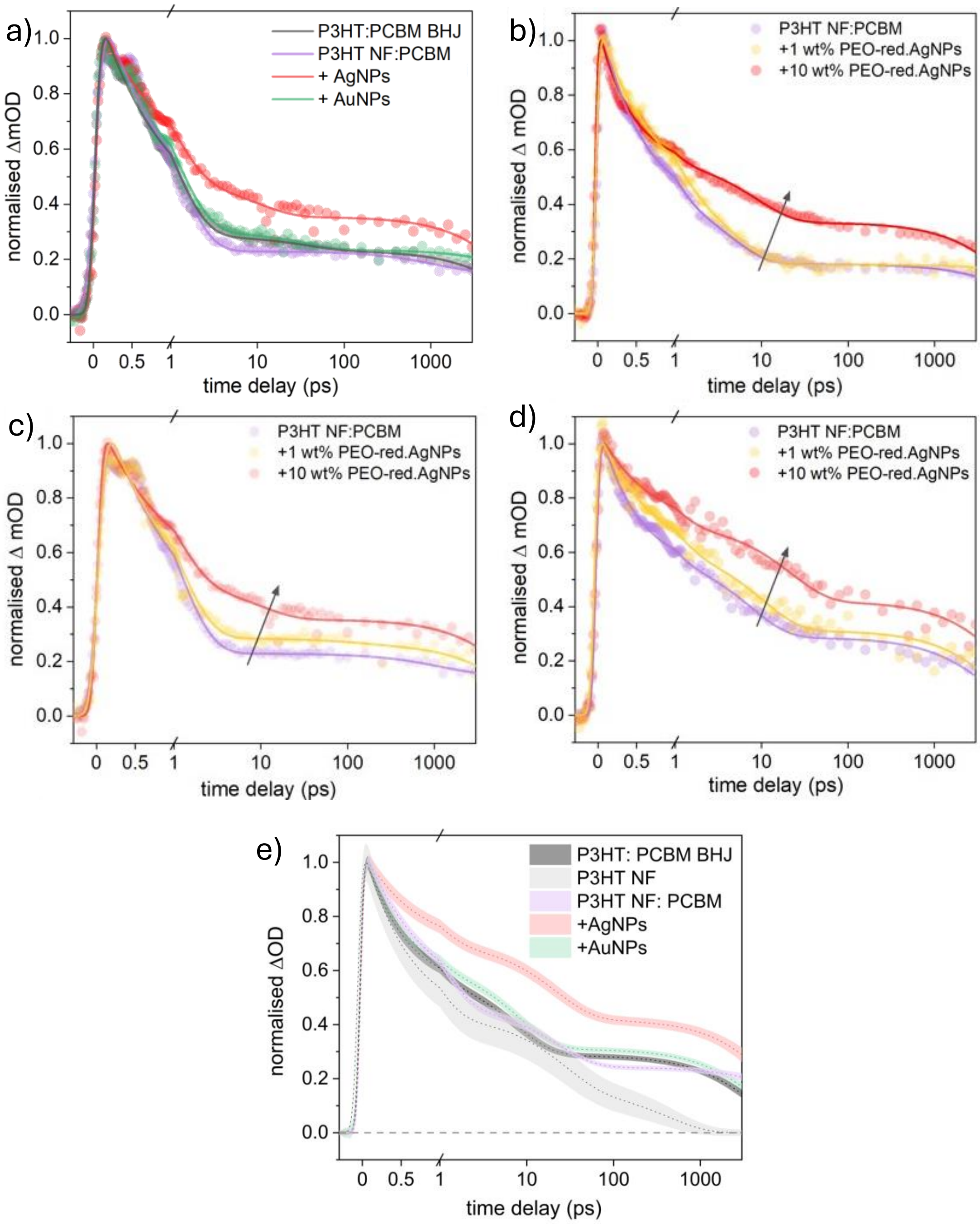

Figure S24 – (a) Relaxation kinetics of the transient polaron pair signal at a probe wavelength of 660 nm, pumped with a 520 nm excitation wavelength. **(b – d)** Decay of **(b)** (0-1) GSB, **(c)** 660 nm polaron pair and **(d)** 950 nm polaron signal presented by pristine and P3HT/AgNP NFs, containing low (1.75 wt% $AgNO_3$ wrt PEO) and high (17.5 wt%) NP loadings. As polaron pairs dominate the signal at 660 nm, this indicates that their lifetimes are extended upon AgNP addition. **(e)** Transient decay profiles at 950 nm probe wavelength showing the standard error in ΔOD derived using Monte Carlo simulation (n=1000) to propagate error determined for fitting parameters.

Supporting Information

Discussion

Signal relaxation is fitted using Equation S6, a tri-component exponential decay function, with an addition parameter to describe the initial photoexcitation.

$$x = y_0 + \sum_{n=1}^{3} A_n\, exp(\frac{\left(\frac{G}{1.655109}\right)^2 - 4\tau_n(x - x_0)}{4\tau_n^2})(1 - \text{erf}\,(\frac{(\frac{G}{1.655109})^2 - 2\tau_n(x - x_0)}{2(\frac{G}{1.655109})\tau_n}))$$

Where A_n is the amplitude and τ_n is the kinetic rate constant in ps of the nth component. G represents the instrument response time in ps, erf is an error function, x is the time delay, and x0 and y0 represent the initial signal magnitude and time delay, respectively. Where y_0 and x_0 is the initial signal prior to photoexcitation, G represents the instrument response time, ~100 fs. The standard error of parameters derived from fitting are applied to the decay profiles in Figure S24e by Monte Carlo simulation (n=1000).

To estimate polaron populations with respect to the initial exciton population, the ΔmOD signal, at a probe wavelength of 950 nm, was taken at its maximum (40 – 200 fs after the initial photo-excitation) and after 1 ns (beyond the temporal regime of charge separation). At this time delays, the transient signal is predominantly ascribed to excitons and polarons, respectively. Okhita et al. estimated the molar absorption coefficients of excitons and polarons by measuring the TAS signal at low and high excitation intensity.[1] At low fluence, signal was ascribed to excitons and at high fluence, signal was predominantly from polarons due to bimolecular recombination. This study determined ε_P at 1000 nm to be comparable to ε_{EX} at 1200 nm and at a 950 nm probe wavelength, we estimate that ε_P is 1.97x that of ε_{EX}. Hence, the transient signal at a single wavelength can be translated into photoexcited species concentrations. Standard errors are propagated during the calculation of polaron populations to produce the uncertainty values given in Table 1.

References

[1] J. Guo, H. Okhita, H. Benten, S. Ito, *J Am. Chem. Soc.* 2015, **131**, 16869.
